# Bivalent impact of social networks on overarming: Insights on the alignment between social and individual interests

**DOI:** 10.1126/sciadv.aed3904

**Published:** 2026-06-03

**Authors:** Feng Fu, Michael Herron, Daniel Rockmore

**Affiliations:** ^1^ Department of Mathematics, Dartmouth College, Hanover, NH 03755, USA.; ^2^ Department of Biomedical Data Science, Geisel School of Medicine at Dartmouth, Lebanon, NH 03756, USA.; ^3^ Program in Quantitative Social Science, Dartmouth College, Hanover, NH 03755, USA.; ^4^ The Santa Fe Institute, Santa Fe, NM 87501, USA.

## Abstract

Drawing on evolutionary game theory, we present a stylized model of gun acquisition for individuals in a society where all have the right to bear arms. In our dynamic model, pairwise confrontations with attendant payoffs take place between individuals who are either armed or unarmed. Individual payoffs depend on the probability of confrontation, the choice to arm, and others’ choices. We show that the likelihood of confrontation affects the optimal societal arming rate and the arming rate that occurs in equilibrium. The latter rate quickly surpasses the former one as the probability of confrontation increases, a phenomenon we call “overarming.” This reflects a misalignment of individual and societal interests. We further show that spatial structures can exacerbate overarming, especially when individuals perceive a deteriorating social environment. Results from our base model and its extensions highlight the importance of understanding how fundamental behavioral dynamics and network heterogeneity influence individual decisions to acquire firearms, informing empirical research for mitigating overarming.

## Teaser

Evolutionary game theory reveals why overarming persists and how individual and social interests can align.

## INTRODUCTION

The right to “bear arms,” codified in the United States by the Second Amendment to the Constitution, presents the vast majority of American citizens with a choice: to exercise this right or not. A 2023 Pew Research Center study found that 30% of adults in the United States own a gun and that 42% live in households with at least one gun, evidence that many people perceive a net benefit to gun ownership (*1*). As of January 2026, in 16 states, the fraction of households with guns was greater than one-half (*2*). Perhaps a more notable statistic is that, as of 2026, the number of guns owned per 100 people in the United States, 120, is almost four times that of its large, industrialized neighbor (Canada) or the country with greatest per capita gun ownership in Europe (Austria) (*3*).

While there are a range of reasons that might lead an individual to purchase a firearm, surveys show that a desire for self-protection is the chief such reason. Gun ownership has been linked to feelings of safety and control in a world perceived to be dangerous (*4*–*7*). Other scholarship on gun possession focuses on effects of top-down regulations (*8*), social norms (*9*), and historical dependence (*10*–*12*) as well as on the psychological processes that link exposure to guns with heightened perceptions of aggression and danger (*13*–*16*). Psychological dynamics surrounding gun ownership in the United States have evolved against a backdrop of shifting cultural narratives and media landscapes, where the emergence of “Gun Culture 2.0” is centered on self-protection, self-defense, and concealed carry (*17*, *18*).

In addition to potential and perceived benefits of gun ownership, there are also costs to possessing firearms. Many choose not to own guns, and reasons to eschew gun ownership include the increased likelihood of fatal self-harm and home violence (*19*, *20*), accidental injuries (*21*, *22*), and suicide (*23*).

The cost-benefit calculus surrounding gun ownership extends to the societal level. Prior studies on the broader consequences of having guns in society have focused primarily on the relationship between gun ownership and various social outcomes (*24*). These include the prevalence of firearm violence (*25*) and crime rates (*26*–*29*). Literature has also identified a high correlation between rates of gun ownership and rates of gun-related homicides (i.e., gun-related deaths not attributed to self-protection) (*30*, *31*). These include “mass shootings” (*32*), whose rate of incidence in the United States [with a high of 689 in 2021; (*33*)] is far and away the highest in the world (*34*).

A world in which high levels of gun ownership are associated with high levels of gun violence suggests that regardless of one’s personal feelings about the value of gun ownership, people considering gun ownership may be making decisions that while individually rational may be collectively suboptimal (with respect to a given level of societal aggression—more on this below), leading to what we describe as an “overarmed” society. Conversely, a society could, in principle, also be suboptimal in its collective decision-making and be “underarmed.”

An overarmed society is one in which its members suffer collectively from the presence of an excessive number of firearms even though, at the individual level, personal decisions regarding gun ownership are rational. We see overarming as a social dilemma with public health implications, and our research strategy, which draws on evolutionary game theory, offers insights on the gun-buying decision-making process that we hope will catalyze an emergent line of inquiry for future research on effective interventions and policy-making aimed at diminishing the level of gun-related violence in American society.

In particular, our contribution to the subject of gun ownership is the development of a model based on evolutionary game theory. The model focuses attention on how any individual’s gun purchasing decision is embedded in similar decisions made by other members of society. In the model, individuals considering owning guns recognize that they will, in the future, encounter other members of society who have also mulled over the matter of acquiring firearms and may in fact own guns. Underarming does not occur in equilibrium in our model (see Results), and hence, we focus on overarming.

Our work is a part of, but different from, the body of work in mathematical modeling of gun violence. There is ongoing debate surrounding data-driven research on this critical issue (*8*, *35*, *36*), and thoughtful mathematical modeling can help tease apart the complex cause-and-effect relationships between gun ownership and adverse societal outcomes that are at the heart of an ongoing and often contentious national conversation in the United States. For example, behavioral models have been used to clarify the role of the use of guns in self-defense as a deterrent of crime (*27*, *29*, *37*), while a mathematical analysis of gun ownership suggests that increased gun availability leads to a higher rate of firearm-related homicides (*31*). In a different direction, recent network contagion models of gunshot-related violence and injuries offer insights into the development of effective targeted policies and interventions intended to reduce firearm-related injuries and deaths (*38*–*42*).

In recent years, evolutionary game theory has proven to be a powerful tool for modeling human behavior, and individual decision-making in particular, in a broad range of social dilemmas (*43*, *44*), including the tragedy of the commons (*45*, *46*). We build on this tradition, developing a game theoretic model that frames individual gun ownership decisions in terms of a small number of parameters. To study the bottom-up behavioral dynamics of gun acquisition, our model accounts for individual payoffs as well as the externalities that stem from the consequences that a member of society imposes on others by virtue of being armed.

Our base model is quite general, framing a society of interacting agents, whose individual actions are antagonistic in nature, seeking advantages over each other. More specifically, our model incorporates what we call a risk of provocation, or confrontation, that can occur in any interaction between two people in a society (*47*). The risk of provocation can be interpreted as a proxy for the level of aggression in a society. Through our analysis, we demonstrate that given a “provocation rate,” overarming—defined formally as when the equilibrium rate of gun ownership in a society exceeds the socially optimal rate of gun ownership—can occur regardless of individuals’ intrinsic preferences for arming or, in contrast, for disarming. It is notable that, for a given provocation rate, our model shows that the social optimum of gun ownership may not necessarily align with the individual optimum.

Our research is inspired in part by the large body of work related to the strategies of nuclear arms in the context of nation-state conflict (*48*). As in that body of work, we identify at the level of individuals something akin to an arms race. Conversely, insights from our current model offer potential extensions to the study of disarmament strategies among nations. In particular, our results highlight a social dilemma of gun ownership wherein what is best for the individual can be at odds with what is best for society as a whole. As such, the work intersects with the wealth of literature on the interactions between individual rationality and societal desiderata; examples include studies of Braess’s paradox and various public health strategies (*49*).

Further recognizing that interactions between individuals are not uniformly random but exquisitely structured (*50*–*53*), we include analyses that incorporate spatial characteristics, both in the form of mathematical stylized representations as well as observed social networks drawn from literature. By incorporating population structures in our modeling, we provide a more fine-grained understanding of the network factors that contribute to the social dilemma of overarming. Unexpectedly, with the addition of spatial structure, we found a “double-edged sword” effect of population structure. Specifically, a lattice population structure (both in theory and simulation) inhibits gun acquisition at a higher critical degree of perceived social deterioration than a well-mixed population. However, it can also result in greater overarming relative to well-mixed populations, particularly at higher provocation rates. This dual impact arises from the interplay between the perceived social payoff structure for arming choices and an underlying network structure that fosters an assortment of like-minded individuals with similar strategies, whether for gun ownership or not.

Last, our work offers analytical intuitions that quantify the conditions under which, despite the risks of gun ownership, a fear of being disadvantaged by being without a gun in a confrontation pushes individuals to arm themselves, thereby leading to overarming, particularly in network populations. In the network setting, we also find an interesting “degree effect” in which a network’s relatively central individuals are less inclined toward gun ownership than those occupying periphery nodes. In combination, this shows that network heterogeneity can act as an amplifier of gun acquisition. Our results reveal the double-edged sword effect of social network structure and have real-world implications for developing strategies for reducing overarming.

## RESULTS

Any analysis of individual arming decisions must account for purely individual reasons to own guns (e.g., for use in hunting) and the cost of possible confrontations that are ubiquitous in society and, depending on others’ arming decisions, can involve guns (e.g., fear of a confrontation with an armed neighbor). Our base model explicitly takes these factors into account within a frequency-dependent setting, across several spatial and social networks, and it illuminates the dynamical implications of misalignment between individual and social interests. Extensions that account for negative-sum payoffs for all confrontations and behavioral heterogeneity in gun use can be found in the Supplementary Materials, but the basic ideas and dynamics in these extensions remain qualitatively unchanged from what we present here.

When an individual buys a gun (a decision we denote by *A*), she receives a benefit *b_g_* and pays a cost *c_g_*. An individual not buying a gun (a decision we denote by *B*) neither receives *b_g_* nor incurs *c_g_*. The sign of the difference *b_g_* − *c_g_*, which we treat as a constant over all individuals in society, characterizes an individual’s preference over guns. For clarity, we focus on the scenario of costly individual gun ownership in our base model (*b_g_* − *c_g_* < 0); other scenarios can be analyzed analogously, as shown in Materials and Methods and the model extensions in the Supplementary Materials.

Beyond individual costs and benefits from gun ownership, our model includes social payoff components that result from pairwise confrontational encounters. These encounters, determined by the provocation rate *p_a_*, are inevitably negative when they occur and may involve guns. The payoff structure for these underlying confrontational encounters, including *AA*, *AB*, and *BB* pairs, determines the collective outcome of arming choices in a well-mixed population, as given in Eq. 1 below

$$\begin{array}{cc} & A\;\;\;B\\ \begin{array}{c} A\\ B \end{array} & \left(\begin{array}{cc} -\delta_{s} & \delta_{e}\\ -\delta_{e} & -\delta_{n} \end{array}\right) \end{array}$$ (1)

*AA* and *BB* interactions are symmetric as they involve two gun owners or two individuals without guns, respectively. An *AA* encounter leads to the payoff vector $\left(-\delta_{s},-\delta_{s}\right)$, where δ*_s_* denotes the cost of a potential shootout (“*s*”) between gun owners. In contrast, the *BB* encounter yields the payoff vector $\left(-\delta_{n},-\delta_{n}\right)$, where δ*_n_* denotes what we call the normal dispute cost (“*n*”) not involving guns. One can think of δ*_n_* as the cost of one unarmed individual arguing with another unarmed individual. The asymmetric *AB* (and, similarly, *BA*) interaction leads to zero-sum payoff vectors $\left(\delta_{e},-\delta_{e}\right)$, where the δ*_e_* is a concession or exploitation cost (“*e*”). This cost can be thought of as the cost of being disadvantaged without a gun when an individual confronts a member of society who owns a gun.

While our approach is primarily theoretical, the assumptions and payoff structure of the model are informed by a broad empirical literature on gun ownership and interpersonal social aggression. The provocation rate *p_a_* in our model captures perceived threat and environmental risk, supported by surveys showing fear of violence and exposure to firearm threats (*47*, *54*, *55*). The individual payoff components, including benefit *b_g_* and cost *c_g_* of gun ownership, reflect common motivations such as protection, recreation, and deterrence, as well as well-documented risks including accidental injury, suicide, and legal liability (*20*–*22*). The structure of social payoffs in confrontational encounters (δ*_s_*, δ*_e_*, δ*_n_*) is similarly grounded in research on the lethality of armed conflict, the escalation risks when both parties are armed (*56*), and the disadvantages faced by unarmed individuals when confronting armed opponents (*57*, *58*). As shown in table S3, these empirically informed model parameters allow us to explore how behavioral dynamics and subjective fear of being disadvantaged interact to produce the collective phenomenon of overarming from a game-theoretical perspective.

**Fig. 1.**
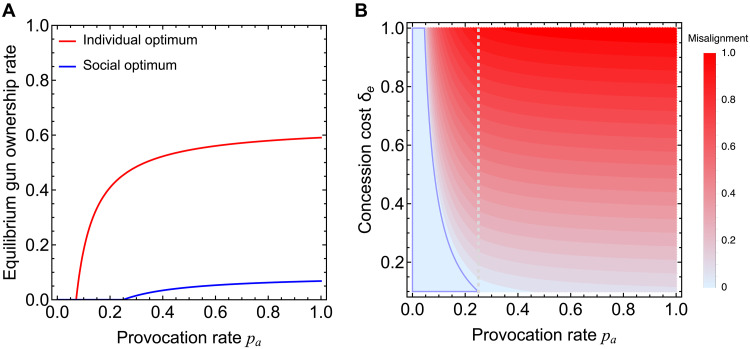
Social dilemma of overarming in well-mixed populations. (**A**) We see a misalignment in arming between the individual self-interest optimum and the social (population) optimum. There is a critical threshold level of the provocation rate *p_a_*, beyond which individuals find it sensible to arm, producing a transition from zero to nonzero gun acquisition (red curve). The socially optimal gun acquisition rate (blue curve) has a greater critical threshold of *p_a_*, rising less steeply than the individual gun acquisition rate in equilibrium. (**B**) Here, we display the effects of varying the provocation rate *p_a_* and concession cost δ*_e_*. The former can be viewed as a proxy for the degree to which a society is dangerous and the latter for the degree of disadvantage borne by individuals when lacking a gun but nonetheless in a pairwise conflict. Misalignment between individual and population gun acquisition rates increases in both *p_a_* and δ*_e_*. The blue region indicates parameter combinations that result in zero gun acquisitions, and the vertical, dashed line marks the critical *p_a_*, below which the social optimum has zero gun acquisitions. Parameters: $b_{g}=0.1$, $c_{g}=0.15$; $\delta_{s}=1$, $\delta_{e}=0.6$, and $\delta_{n}=0.1$ (A); $\delta_{s}=1$ and $\delta_{n}=0.1$ (B).

We model the spread of gun acquisition as an evolutionary dynamics phenomenon (see Materials and Methods). In particular, this helps us characterize individual and social determinants of overarming in a well-mixed population (Fig. 1) without the complications of network structure. In a well-mixed population, whether *A* or *B* performs better depends on their frequencies in the population, so the relevant comparison is in “expected payoffs,” which average over all possible encounters between *A* and *B*. This is precisely why the strategic incentives of gun ownership are frequency-dependent: When guns are rare, arming is attractive because it yields an advantage in *AB* encounters while *AA* encounters are unlikely; in contrast, when guns are common, additional arming mainly increases exposure to costly *AA* encounters, thereby lowering expected payoffs. This frequency-dependent mechanism can lead to a coexistence equilibrium, as shown in Fig. 1.

In Fig. 1, we see the results for the fully mixed interaction model. The rate of gun ownership in equilibrium exhibits a transition from zero to nonzero at the critical value $p_{a}^{*}=\left(c_{g}-b_{g}\right)/\left(\delta_{n}+\delta_{e}\right)$. When $p_{a}<p_{a}^{*}$, in equilibrium, no member of society owns a gun. This is what one might call universal disarmament. As the provocation rate *p_a_* increases, the transition to a society in which some members own guns reflects how the perceived degree of environment deterioration (for which the probability of confrontation is a proxy) affects arming choices from the bottom up. From the perspective of a central social planner wanting to minimize total societal costs associated with guns, the population optimum rate of gun acquisition for society not only is significantly smaller than the equilibrium rate, but the former also requires a notably higher critical level of *p_a_* to induce a population optimum gun ownership rate that is greater than zero (Fig. 1A). From the perspective of a social planner, there is greater resistance to the acquisition of guns. The misalignment between individual incentives that operate in equilibrium and what is optimal for society as specified by a social planner gives rise to overarming.

We can use the level of misalignment between an equilibrium rate of gun ownership and the socially optimal rate to quantify the severity of overarming. Overarming is aggravated by increases in the concession cost δ*_e_* as well as in the provocation probability *p_a_*, as shown in Fig. 1B. In contrast, misalignment is reduced with increases in the cost of shootout δ*_s_* and in the cost of a dispute δ*_n_* between unarmed individuals (see fig. S1). These results can be interpreted as implying that key determinants of the extent to which a society is overarmed are the degree of perceived social deterioration and the fear of being disadvantaged in a confrontation without a gun.

The analytical prediction of our game theoretic model qualitatively mirrors empirically observed trends. For example, the threshold behavior in gun acquisition as provocation increases (Fig. 1) reflects survey evidence that fear of being disadvantaged is a major motivator of firearm purchase (*11*, *18*). The role of the concession cost (δ*_e_*) in shaping overarming aligns with qualitative findings that individuals perceive themselves as vulnerable when others are armed, leading to preemptive gun ownership (*13*, *54*).

With this reference to pairwise (individual-to-individual) interactions, our model operationalizes observed behavioral responses within a proof-of-concept framework that extends to the characterization of social network effects. Actual populations are not well-mixed—this is one interpretation of the high clustering coefficient generally observed in social networks [see, e.g., (*59*)]—and any specific kinds of mixing patterns (“spatial populations”) will have a strong effect on dynamics that depend on interactions. For example, social network structure is known to affect behavioral spreading dynamics in which social influence plays a significant role (*60*).

**Fig. 2.**
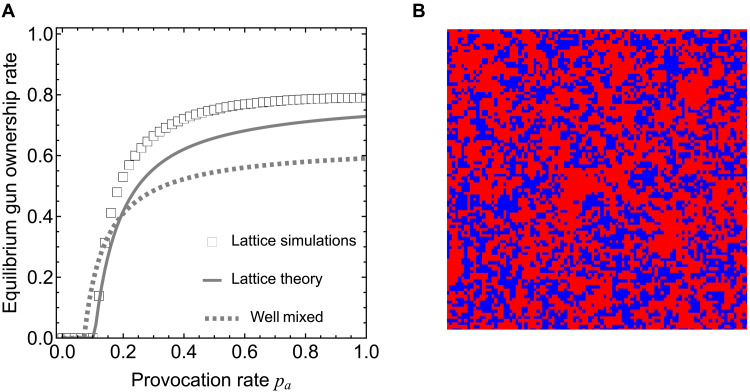
Social dilemma of overarming in spatial (square lattice) populations. We consider the evolutionary dynamics of arming versus disarming on a square lattice with the von Neumann neighborhood. (**A**) Compared to a well-mixed population (dotted line), spatial structure is a double-edged sword, yielding a higher provocation rate threshold for arming but, when the provocation rate is large, precipitating a greater degree of overarming. The squares represent simulation results, and the solid line represents analytical predictions using the pair approximation method. (**B**) Spatial snapshot of individuals’ arming choices where red denotes acquisition of a gun and blue the opposite. Peer influence leads to clustering of like-minded individuals with similar arming choices. Parameters are based on a population of size 100 × 100: $b_{g}=0.1$, $c_{g}=0.15$, K=0.1, $\delta_{s}=1$, $\delta_{e}=0.6$, and $\delta_{n}=0.1$. For (B), $p_{a}=0.16$. Simulation results in (A) are averaged over 100 independent runs.

We start with spatial populations wherein individuals are situated on a regular spatial lattice. This is a standard first spatial model for encapsulating common characteristics of local interactions often observed in community neighborhoods. We run stochastic, individual-based simulations to determine equilibrium fractions of gun acquisitions and compare them with the analytical results obtained by using the pair approximation method (*61*). Our analytical predictions are consistent with simulation results. In particular, we show that there exists a critical threshold of *p_a_* above which the equilibrium rate of gun acquisitions is strictly positive (Fig. 2A). As shown in Fig. 2, spatial population structure inhibits the onset of gun acquisitions compared to well-mixed populations. The critical value of *p_a_* that induces positive rates of gun ownership is lowest for a well-mixed population.

A spatial population structure can facilitate the clustering of like-minded individuals with similar arming choices. This is a prominent and intuitive feature of our model, the consequences of which can be seen in Fig. 2B. This clustering in conjunction with the crossover between the curves in Fig. 2A highlight a “double-edged sword” phenomenon particular to spatial populations, namely, in societies with low levels of confrontation (*p_a_* close to zero), clustering can depress gun ownership rates in comparison to rates from a well-mixed population. However, in societies with high levels of confrontation (*p_a_* much greater than zero), the opposite obtains.

**Fig. 3.**
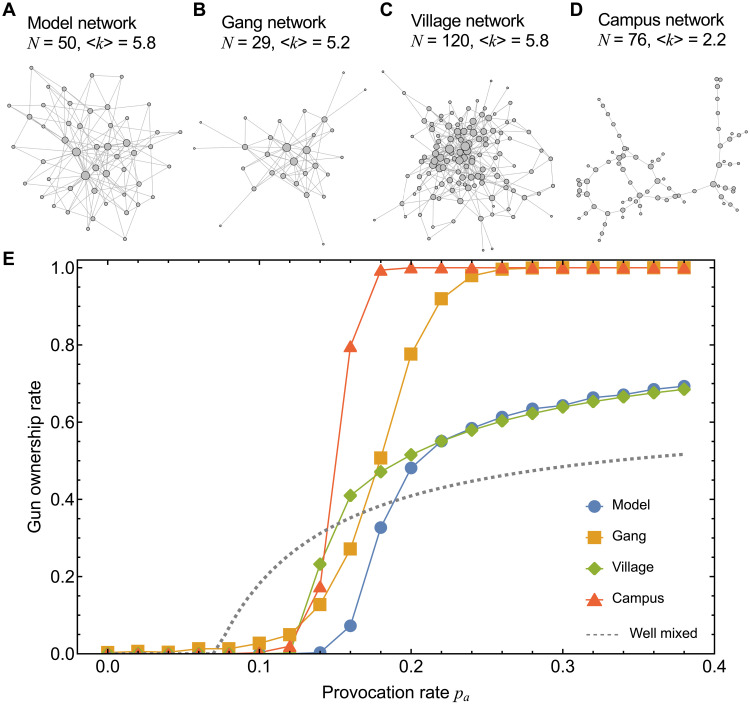
Social network structure impacts arming choices and the social dilemma of overarming. We consider the evolutionary dynamics of arming choices in four kinds of networks: (**A**) a model degree-heterogeneous network, (**B**) a gang network (*62*), (**C**) a village social network (*65*), and (**D**) a campus network (*63*, *64*). Networks in (B), (C), and (D) are actual social networks with different contexts. (**E**) Comparison of gun acquisition rates in equilibrium as a function of the provocation rate *p_a_*. The model network and the village network both show transitions similar to those in the spatial lattice population depicted in Fig. 2A. However, the topological characteristics of the gang and campus networks appear to more closely resemble stylized structures such as star-like graphs, thereby greatly amplifying gun acquisition rates. Parameters: K=0.1, $b_{g}=0.1$, $c_{g}=0.15$, $\delta_{s}=1$, $\delta_{e}=0.6$, and $\delta_{n}=0.1$. Simulation results are averaged over 2000 runs.

Moving from well-mixed and lattice population models to actual social networks, we now consider the dynamics of gun acquisition on observed social networks from developed regions (*62*–*64*) and developing regions (*65*), some of which have been plagued by gun-related violence (Fig. 3, B to D) (*66*, *67*). To this end, we apply our model to various social network topologies documented by other scholars so that our simulation results reflect observed structural heterogeneity in real-world environments.

Our real-world population structures illustrate network heterogeneity: Some individuals may be in a more central network position (i.e., social hubs) compared to others (e.g., peripheral nodes) (Fig. 3, B to D). Figure 3E shows the critical value of *p_a_* that induces positive gun ownership across the three real social networks, revealing an aggregate pattern of transitions from zero to nonzero gun acquisition as the provocation rate *p_a_* increases (also see fig. S3). Using a model network as the baseline (Fig. 3A and fig. S2), our three heterogeneous networks show similar critical thresholds of *p_a_*, comparable to those given by the spatial lattice population, and therefore, they invariably manifest the aforementioned double-edged sword effect as well.

That said, there is a noticeable difference in the subtle curvatures that characterize the exact transitions: The village network appears to more closely resemble the model network, whereas the gang and campus networks are distinct from the others in that the latter have motifs—star-like subgraphs—in which a central node is connected to a few periphery nodes (Fig. 3, A to D). Our simulations confirm that these transitions in gun acquisition, characterized by all-or-nothing shifts from universal disarmament to universal armament, are largely attributed to these stylized structures (fig. S3). Hence, our results provide insights into the impact of extreme network heterogeneity such as those exhibited by star-like graphs.

Now, we turn to intuition behind how an individual’s network position affects the decision to own a gun. A central (high-degree) node benefits from gun ownership (*A*) when neighboring periphery nodes do not own guns (*B*). However, the advantage of gun ownership received by a center node can quickly reverse itself when gun ownership spreads to periphery nodes. When this happens, the expected gun ownership (*A*) payoff for a center node can drop below that of choosing not to own a gun (*B*). In this way, a central node (individual) who buys a gun can start a process that leads to the node’s not wanting to own a gun. This type of negative feedback can result in the intriguing degree effect we observe on heterogeneous social networks: Even with just a few neighbors, the central nodes in a network are less inclined to own guns than their periphery counterparts (fig. S2). This theoretical result is in accord qualitatively with network studies of gunshot injuries showing that central individuals are more likely to be victims of gun violence (*38*). In other words, it is in the self-interest of central individuals to reduce the inclination to acquire firearms.

Beyond simulations, our analytical results on star graphs (figs. S4 and S5) highlight the subtlety inherent in how network position affects individual (and self-interested) arming decisions. Overall, we find that network heterogeneity can be considered an amplifier of gun acquisition and can greatly aggravate overarming (Fig. 3 and figs. S3 and S4).

**Fig. 4.**
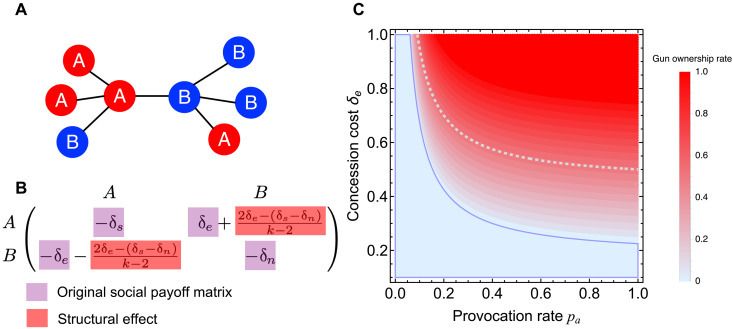
Population structure can significantly transform underlying payoff structures, especially by exaggerating the fear of losing out. (**A**) Intuition for how population structure can increase overarming. Compared to a well-mixed population, peer influence leads to clusters of individuals who own guns (*A*) and clusters of individuals who do not own guns (*B*). Theory shows that, on average, an *A* individual has one more *A* neighbor than a *B* individual. Spatial equilibrium is achieved at the boundary between *A* and *B* when these two types have equal expected payoffs. (**B**) How spatial structure transforms the effective social payoff matrix associated with gun ownership, in particular manifested through the antidiagonal elements, which have the potential to augment the fear of losing out. (**C**) Characterization of the payoff structure conditions that can cause the double-edged sword effect for gun ownership in spatial populations. The spatial equilibrium of gun ownership, displayed as a heatmap, is a function of the provocation rate *p_a_* and the concession cost δ*_e_*. The blue region reflects model parameter combinations resulting in a gun ownership rate of zero (compare with Fig. 1B), and the dashed line marks the boundary above which the double-edged sword effect can occur. Closed-form analytical results are obtained on a square lattice with degree *k* = 4 and under weak selection limit, K≫1. Payoff parameters are as in Fig. 1B.

Further analytical insights explicate the dual impact of population structure on gun ownership and its potential implications for mitigating overarming. Without loss of generality, our theoretical analysis focuses on the limiting case, K≫1, and in this limit, we obtain closed-form results for the local densities of gun acquisition choices in the network, such as $q_{A|A}$ and $q_{B|A}$ (see details in the Supplementary Materials). Because of clustering/assortativity effects, on average, the number of gun-owning neighbors of an individual gun-owner (*A*) is one more than the number of gun-owning neighbors of a non–gun owner (*B*), namely, $\left(k-1\right)\;\left(q_{A|A}-q_{B|A}\right)=1$ (Fig. 4A). Using this result, we can derive the network equilibrium of gun acquisitions in closed form. Our results show that population structure transforms the individual and social payoff of gun ownership. Regarding the latter, the penalty associated with being disadvantaged by not owning a gun δ*_e_* becomes $\delta_{e}+\left[2\delta_{e}-\left(\delta_{s}-\delta_{n}\right)\right]/\left(k-2\right)$ in network populations, assuming the network degree k≥3 (see Fig. 4B). This analytical result helps us intuitively understand the interplay between population structure and the underlying payoff structure.

In light of the demonstrated regularity between exaggerated fears and firearm purchases (*47*), our analytical result (Fig. 4C) suggests that perhaps a public information campaign that helps individuals to realistically estimate the concession cost δ*_e_* associated with threat perception could dampen the tendency for societies to suffer from overarming. When the fear of being disadvantaged δ*_e_* drops below a critical threshold that depends on the perceived environmental safety (marked by the dashed line in Fig. 4C), the extent of overarming becomes less pronounced compared to a well-mixed population. In theory, it is even possible to induce the networked population to universally cease purchasing guns, leading to universal disarmament, especially when the cost δ*_e_* falls below the threshold $\delta_{e}^{*}<c_{g}-b_{g}+\left[\delta_{s}-\left(k-1\right)\delta_{n}\right]/k$ (Fig. 4C and compare with Fig. 1; see the Supplementary Materials for derivation details). This theoretical result provides an intuitive insight into when overarming is most likely and when universal disarmament is possible.

This type of understanding could be useful for conversations surrounding the matter of gun control, illustrating how our model and its applications to networked populations have practical implications for conceptualizing gun control measures grounded in the relationship between network structure and individuals’ fears about concession.

Psychological studies underscore how the presence of guns can, as a threat response, trigger the acquisition of more guns (*4*, *5*), reflecting psychological processes that link prevalence of guns with heightened perceptions of danger and societal aggression (*6*, *13*–*18*). This leads to a self-reinforcing feedback loop involving gun ownership and the perceived risk of being near individuals who own guns.

**Fig. 5.**
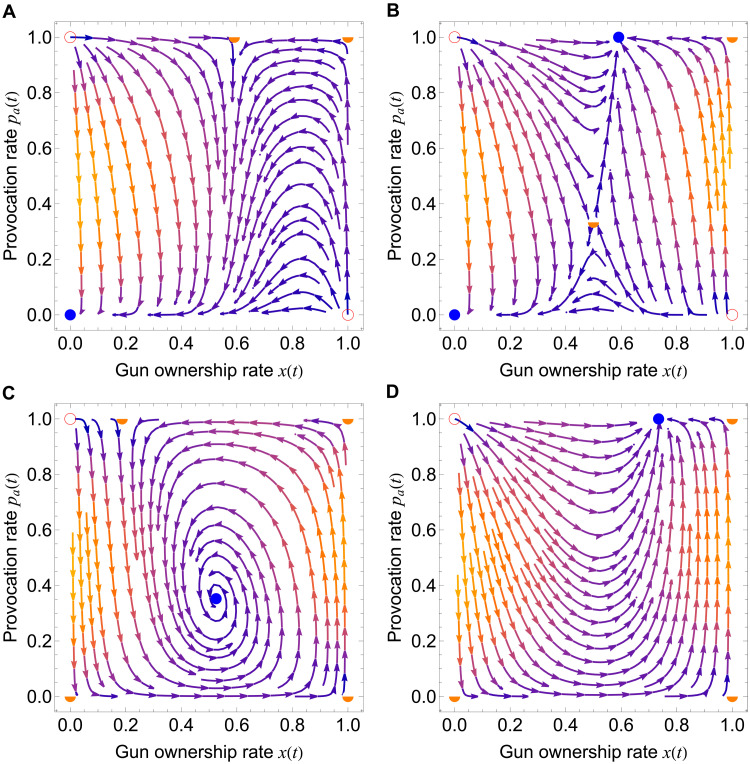
Coevolutionary dynamics of the provocation rate *p_a_* and the gun acquisition rate, denoted here by *x*. Shown are streamplots of the time-evolution trajectories of *x*(*t*) and *p_a_*(*t*) along with their possible equilibria (stable nodes, filled circles; unstable nodes, empty circles; saddle point, half-filled circle) for cases (**A**) and (**B**), where $b_{g}-c_{g}<0$, versus cases (**C**) and (**D**), where $b_{g}-c_{g}>0$. (A) and (B) demonstrate the impact of varying the parameter θ, while (C) and (D) show the impact of varying the parameter δ*_e_*. Parameters for (A) and (B): $b_{g}=0.1$, $c_{g}=0.15$, $\delta_{e}=0.6$, $\delta_{n}=0.1$, and $\delta_{s}=1$, with θ=0.2 in (A) and θ=1 in (B); for (C) and (D): $b_{g}=0.8261$, $c_{g}=0.6417$, $\delta_{n}=0.0012$, $\delta_{s}=1$, and θ=0.9037, with $\delta_{e}=0.0015$ in (C) and $\delta_{e}=0.55$ in (D).

Unlike our base model, wherein the provocation rate *p_a_* is exogenously prescribed and remains fixed, we now introduce an extended model wherein *p_a_* represents a subjective threat perception arising from the interplay between self-serving gun acquisition and perceived threats. This subjective perception of the confrontation rate *p_a_* plays an important role in the deliberation of arming choices (see detailed analysis in the Supplementary Materials). For a well-mixed population, we have

$${\dot{p}}_{a}=p_{a}\;\left(1-p_{a}\right)\;\left[\theta \;x-\left(1-x\right)\right]$$ (2)

where the new parameter θ quantifies how strongly individuals respond to others’ gun acquisitions. The perceived risk is counteracted by the fraction of the population that does not own guns, 1 − *x*. As shown in Fig. 5, for a population perceiving the individual payoff of owning a gun as negative, a small θ can lead to complete disarmament (Fig. 5A). For large θ, the population exhibits bistable dynamics (Fig. 5B): Although zero gun acquisition is still possible, the population can also converge to maximum levels of perceived risks and high gun acquisition (compare with Fig. 1, with all other payoff parameters equal except for the sensitivity parameter θ).

Combining our extended coevolutionary model with data on gun sales in the United States, which peaked during the COVID-19 pandemic, the best estimates of model parameters (see table S2) suggest that, particularly in the context of American gun culture, the individual benefit of gun ownership ($b_{g}\approx 0.83$) exceeds its cost ($c_{g}\approx 0.64$), aligning with survey data indicating that most owners report a net sense of safety and utility from firearm possession (see table S3). The social cost of shootouts is fixed at $\delta_{s}=1$, allowing us to quantify the relative magnitudes of concession costs ($\delta_{e}\approx 0.0015$) and unarmed disputes ($\delta_{n}\approx 0.0012$). The model-fitted value of θ≈0.90 indicates that individuals strongly respond to others’ gun purchasing decisions, triggering significant threat responses and resulting in oscillatory dynamics (Fig. 5C). The quantitative ranking $\delta_{s}\gg \delta_{e}>\delta_{n}$ reflects the common perception that the harm from yielding in an armed encounter or from normal disputes is minimal relative to the potentially fatal consequences of gun violence. Moreover, individuals fear being disadvantaged without a gun more than they fear unarmed confrontations (i.e., $\delta_{e}>\delta_{n}$).

Our extended model aligns with the broader context of American gun culture, emphasizing how perceived risks and gun ownership coevolve in a self-reinforcing cycle. All else being equal, a further increase in the fear of being disadvantaged can amplify this self-reinforcing cycle, leading to overarming and persistent perceived risks (Fig. 5D), as similarly shown in Fig. 5B, albeit with *b_g_* − *c_g_* < 0 in the latter. Together, our parameter choices, situated in empirical findings (see the Supplementary Materials for details), highlight how population dynamics and subjective perceptions can coevolve to produce collective overarming.

## DISCUSSION

In this paper, we present a dynamic, evolutionary game-theoretic model of a population in which individuals can choose to acquire guns. Our model’s small set of parameters, which includes the perceived threat of conflict in any person-to-person interaction and the personal danger intrinsic to owning firearms, allows us to characterize externalities associated with individual arming decisions and to compare both socially optimal arming rates and arming rates that occur in equilibrium. Beyond the pairwise interactions considered here, in the Supplementary Materials, we also examine the dynamics of gun acquisition in group settings, as higher-order interactions are relevant in the context of the tragedy of the commons (*53*). Our work offers insights into the widespread gun ownership that can arise de novo from the availability of guns to a population, despite the risks of gun ownership.

Our model includes interactions between members of society, highlighting how the decision to own a gun is not made in isolation but rather is influenced by the choices of others, what can be thought of as individuals’ “contact networks.” We investigate the dynamics of gun acquisitions in both a fully mixed model and over a variety of real social networks, using the pair approximation method to predict equilibrium rates of gun acquisition in spatial populations and to demonstrate that patterns of overarming emerge in real social networks.

The role of network structure in shaping social behavior has been extensively studied in the context of cooperation, trust, and other prosocial behaviors (*50*, *68*), but its effect in the setting of general negative-sum interactions, like firearm acquisitions, is much less well understood, and this is the relevant setting for gun interactions. Through simulating the spread of gun acquisition over time, we investigate the emergence of phenomena like overarming and identify the factors that drive populations toward a suboptimum in which too many of society’s members are armed.

A primary takeaway from our work is the significance of the provocation parameter *p_a_*. We view this as a proxy for the “temperature” of the environment in which our arming game plays out. When confrontations are more likely—higher temperature—overarming is more dramatic. To that end, any efforts spent on lowering the temperature of a loosely regulated gun-owning society, as well as reducing individuals’ sensitivity to others’ gun acquisition (lowering the feedback parameter θ, as shown in Fig. 5), could be helpful in reducing overarming. This is of course a very difficult problem, especially in a highly polarized society (*69*).

Another possible intervention approach suggested by our work comes from the two-sided impact of network structure, which could be redirected toward suppressing gun acquisitions. To be concrete, raising public awareness about the consequences of overarming, coupled with the costs of gun violence, might contribute to mitigating the exaggeration of fears as well as addressing unfounded concerns regarding social safety and threats. In this regard, network heterogeneity can also be harnessed to help close what we have identified as an overarming gap between socially optimal and equilibrium rates of gun ownership.

For instance, due to a negative feedback loop, social centers involved with guns can lead to the demise of their own success, thereby presenting a compelling rationale for choosing not to obtain a gun, a behavior that actually aligns with the interest of social hubs. From this perspective, network-based targeting methods can be optimized for the alignment of individual interests, especially by those in the central network position, and thus reduce the level of overarming. For example, targeted interventions aimed at perturbing the social network through small, strategic network changes can help mitigate overarming (see figs. S6 and S7). Extending the present work by simulating different policy scenarios can help evaluate the impact of various gun control measures on the spread of arming choices and identify the most promising network-based strategies for reducing gun-related violence (*70*).

Our approach does have some limitations, and one of these is its focus on individual-level choices. Although our base model includes a proxy for a general (and fixed) level of provocation (a proxy for general feelings about safety in a society), our results do not fully account for the influence of broader social, economic, and political factors that may feed into the choice to arm. For example, the COVID-19 pandemic led to a surge in firearm sales as a result not only of concerns about personal safety but also about social unrest and uncertainty surrounding the pandemic’s trajectory (*47*). That said, by incorporating gun sales data, our extended coevolutionary model provides insights into understanding self-reinforcing cycles of self-serving gun acquisition as a response to threats and self-fulfilling perceived risks (Fig. 5 and also see fig. S9). Our model also does not account for heterogeneity in individual attitudes toward guns. As it examines arming choices in networks, in principle, the model can be extended to incorporate environmental feedback in order to study the relationship between gun ownership and environmental deterioration in the changing world using eco-evolutionary games (*71*). By further integrating environmental dynamics and feedback mechanisms, our model can provide a more comprehensive understanding of how individual decision-making processes are influenced by the rapidly changing context of social environment and how individuals’ decisions, in turn, affect the broader social and political landscape (*17*).

Moreover, we only consider person-to-person provocative confrontations among citizens, thus taking symmetry of roles as a starting point. It may be plausible to extend our model by introducing subpopulations with asymmetrical roles, such as criminals and victims in the context of firearm use for self-defense (*29*). In addition, variations in gun control policies, the role of interest groups, and the historical context of gun rights in the United States are all factors that contribute to the complex landscape of gun ownership and gun-related violence (*24*). Incorporating these meaningful societal features into a future version of our model and studying the diffusion of gun acquisition and equilibrium states will provide a more comprehensive, mechanistic understanding of overarming.

We believe that our application of evolutionary game theory to arming choices provides a timely and valuable perspective on the complex dynamics driving an important social phenomenon. And with our model, we hope to shift the focus from divisive debates over gun ownership rights to a more comprehensive and evidence-based discussion, highlighting the cost-benefit viewpoint that acknowledges both positive (e.g., recreation) and negative (e.g., related injuries and deaths) effects of firearm ownership. Our model implicitly advocates for a public health approach to gun violence that fosters collaboration among various stakeholders, including policymakers, researchers, and community members (gun owners and non-owners) to better identify the root causes, risk factors, and potential prevention strategies that promote the safety and well-being of society as a whole (*36*).

We acknowledge that our model simplifies the rich and multicausal nature of gun ownership decisions. Political, cultural, and media-related factors, such as those highlighted in recent sociological work (*8*, *17*), also play critical roles. Our aim is not to offer a “one-size-fits-all,” full account of gun ownership and sales trends, but to distill key behavioral and network structural features into a tractable model. The parameters used are not arbitrary; rather, they are guided by established data on gun violence and ownership (see table S3), including the asymmetric risk profiles of armed versus unarmed confrontations (*56*) and the psychological costs of perceived disempowerment (*11*). We view the model as a hypothesis-generating framework, capable of producing testable predictions about how perceived risk and gun ownership coevolve. Future empirical studies can validate or refine these predictions using behavioral experiments or large-scale observational data. Such fine-grained, individual-level data can not only further validate the payoff structure characterizing social aggression dynamics involving guns, but also shed light on individual motivations for gun purchases or abstention, as well as on heterogeneity in gun owners’ propensity to escalate conflicts.

In light of these realistic considerations, we also analyze two model extensions in the Supplementary Materials that relax key assumptions introduced above. First, we consider negative-sum *AB* encounters by discounting the gun owner’s perceived advantage, replacing the *AB* payoff vector with $\left({\alpha \delta }_{e},-\delta_{e}\right)$ for 0 < α < 1. This reduction in the perceived advantage of owning a gun lowers both the individually optimal and socially optimal gun-ownership rates; moreover, the social optimum can drop to zero once α falls below a threshold. However, the misalignment between individual and social interests, and thus overarming, persists throughout the range 0 < α < 1 (fig. S10). Second, we extend the model to include a third behavioral type, fair-use gun owners, who de-escalate in conflicts with non-owners and use a gun only when the opponent is armed and also uses a gun (fig. S11). This addition generates richer three-type dynamics, including damped rock-paper-scissors–like cycling when individual gun ownership is costly, while the coexistence equilibrium highlighted in Fig. 1 can still be sustained across scenarios.

Last, our work presents a theoretical and stylized modeling approach. The model is not empirically validated through behavioral data, and the parameters—although motivated by prior literature (*6*, *13*–*18*) (also see table S3)—are not calibrated to specific psychological measurements or field observations. Instead, our model serves as a conceptual and analytical framework aimed at generating testable hypotheses and revealing potential social and network-structural factors of the overarming dilemma. While our findings are theoretically grounded in evolutionary game theory with simulation results consistent with model assumptions, external validation through behavioral studies, experimental work, or individual-level data analysis is an important next step. As such, our contribution should be viewed as a proof of concept that can guide future interdisciplinary research to empirically test the model’s assumptions and analytical predictions.

To conclude, using evolutionary game theory, our proof-of-concept model offers a unique, bottom-up perspective for understanding the complex issue of overarming, specifically in the context of human behavior and decision-making. By focusing on individual risk perceptions and network interactions that are of negative sum in nature, our study sheds light on the intricate dynamics of overarming. Our approach highlights the importance of accounting for the complexity of individual preferences, social influence, and the role of externalities in shaping arming choices. In doing so, it identifies and addresses a root cause of overarming—fears of being disadvantaged—stemming from the double-edged sword effect of social network structure. Our findings hold meaningful implications for the development of more targeted and effective strategies to tackle the challenges posed by overarming in our societies. We hope that this approach has the potential to inspire further research in this critical area (*72*), leading to network-based interventions that resonate with individual motivations and societal dynamics and ultimately promote public safety and well-being.

## MATERIALS AND METHODS

### Base model for understanding overarming

Here, we use the terms “arming,” “gun acquisition,” and “gun ownership” interchangeably. Although we recognize that there are many different kinds of guns (or “firearms”) of widely varying levels of lethality—such as handguns versus long guns—we do not draw distinctions between these weapons, lumping them all into the class of “gun.”

Owning a firearm incurs an individual cost *c_g_*. This includes resource costs such as those incurred in purchasing or maintaining a gun as well as the costs of potential adverse outcomes, such as those due to self-injuries due to unintentional discharge. The individual benefit of owning a firearm is *b_g_*. This includes utilities from professional and/or recreational uses (e.g., hunting) as well as any perceived psychological benefit as well as the benefits as a deterrent to crime and violence or protection in a confrontational situation (notice that we do not distinguish here between legal and illegal activities). Thus, the difference *b_g_* − *c_g_* gives an individual payoff as a gun owner. Population preferences can be characterized by the sign of *b_g_* − *c_g_*: *b_g_* − *c_g_* > 0 in favor of arming, *b_g_* − *c_g_* = 0 neutral (indifference), and *b_g_* − *c_g_* < 0 in favor of disarming.

Externalities of arming versus disarming depend on how frequently provocations, which may involve guns, occur among individuals. We use the parameter *p_a_* to denote the relative rate of provocation, that is, the incidence that individuals come into disputes, as compared to their everyday normal life. If provocation occurs among two armed individuals, they both receive the same payoff, −δ*_s_* (the cost of a shootout). In a provocation involving an armed individual against an unarmed individual, the former receives δ*_e_* by dominating (outgunning or exploiting) the disarmed individual, who retreats and receives −δ*_e_* as a result. For provocations between two unarmed individuals, both receive −δ*_n_* due to their normal quarrels and brawls. We assume the rank order of provocation costs satisfies δ*_n_* < δ*_e_* < δ*_s_*. Given this payoff structure, the decision landscape connected to provocative interactions with arming versus disarming choices resembles classic snowdrift games [or the so-called Hawk-Dove game; (*73*)] (a negative sum armed-unarmed interaction is explored in the Supplementary Materials, as is a more textured assumption of the armed population).

We use evolutionary game theory to study the population dynamics of arming decisions of individuals. Arming means the decision to be a gun owner, and not arming means the decision not to own a gun. The population equilibrium reflects the long-run percentage of gun ownership. We denote by *x* the percentage of individuals who are gun owners in the population. Assuming a well-mixed population, the expected average payoff *g*_1_(*x*) of arming as a gun owner is

$$g_{1}\left(x\right)=b_{g}-c_{g}+p_{a}\left[-x\delta_{s}+\left(1-x\right)\delta_{e}\right]$$

The expected average payoff of disarming is

$$g_{0}\left(x\right)=p_{a}\left[-x\delta_{e}-\left(1-x\right)\delta_{n}\right]$$

The time evolution of individual arming choices, *x*, can be described by the replicator equation

$$\frac{\mathrm{dx}}{\mathrm{dt}}=x\left(1-x\right)\left[g_{1}\left(x\right)-g_{0}\left(x\right)\right]$$

The population can have a stable interior equilibrium (evolutionarily stable strategy, ESS), x*, which corresponds to the individual optimum by using a mixed strategy x*

$$x^{*}=\frac{b_{g}-c_{g}+p_{a}\left(\delta_{n}+\delta_{e}\right)}{p_{a}\left(\delta_{n}+\delta_{s}\right)}$$

The population equilibrium depends on their intrinsic preference of arming versus disarming, determined by the sign of *b_g_* − *c_g_*. Without loss of generality, we first analyze the case *b_g_* − *c_g_* < 0. The cases for *b_g_* − *c_g_* > 0 and *b_g_* − *c_g_* = 0 can be analyzed analogously.

Assuming *b_g_* − *c_g_* < 0, the population equilibrium (ESS) x* is given by

$$x^{*}=\left\{\begin{array}{cc} 0 & p_{a}\leq \left(c_{g}-b_{g}\right)/\left(\delta_{n}+\delta_{e}\right)\\ \frac{b_{g}-c_{g}+p_{a}\left(\delta_{n}+\delta_{e}\right)}{p_{a}\left(\delta_{n}+\delta_{s}\right)} & p_{a}>\left(c_{g}-b_{g}\right)/\left(\delta_{n}+\delta_{e}\right) \end{array}\right.$$ (3)

The social optimum *x^s^* can be obtained by maximizing the total population payoff (per capita), ${\mathrm{xg}}_{1}\left(x\right)+\left(1-x\right)g_{0}\left(x\right)$

$$x^{s}=\left\{\begin{array}{cc} 0 & p_{a}\leq \left(c_{g}-b_{g}\right)/\left(2\delta_{n}\right)\\ \frac{b_{g}-c_{g}+2p_{a}\delta_{n}}{2p_{a}\left(\delta_{n}+\delta_{s}\right)} & p_{a}>\left(c_{g}-b_{g}\right)/\left(2\delta_{n}\right) \end{array}\right.$$ (4)

In this scenario, both x* and *x^s^* are increasing with respect to *p_a_* (which measures the degree of deterioration in social environment), and the critical threshold for nonzero percentage of gun ownership is $\left(c_{g}-b_{g}\right)/\left(\delta_{n}+\delta_{e}\right)$, which is smaller than the threshold $\left(c_{g}-b_{g}\right)/\left(2\delta_{n}\right)$, above which social optimum is also nonzero. For x* to be greater than *x^s^*, it is required that $p_{a}>\left(c_{g}-b_{g}\right)/\left(2\delta_{e}\right)$, which holds whenever $x^{*}>0$ [$p_{a}>\left(c_{g}-b_{g}\right)/\left(\delta_{n}+\delta_{e}\right)$].

For the scenario with $b_{g}-c_{g}>0$, we have

$$x^{*}=\left\{\begin{array}{cc} 1 & p_{a}\leq \left(b_{g}-c_{g}\right)/\left(\delta_{s}-\delta_{e}\right)\\ \frac{b_{g}-c_{g}+p_{a}\left(\delta_{n}+\delta_{e}\right)}{p_{a}\left(\delta_{n}+\delta_{s}\right)} & p_{a}>\left(b_{g}-c_{g}\right)/\left(\delta_{s}-\delta_{e}\right) \end{array}\right.$$

and

$$x^{s}=\left\{\begin{array}{cc} 1 & p_{a}\leq \left(b_{g}-c_{g}\right)/\left(2\delta_{s}\right)\\ \frac{b_{g}-c_{g}+2p_{a}\delta_{n}}{2p_{a}\left(\delta_{n}+\delta_{s}\right)} & p_{a}>\left(b_{g}-c_{g}\right)/\left(2\delta_{s}\right) \end{array}\right.$$

For $b_{g}-c_{g}>0$, both x* and *x^s^* are decreasing with respect to *p_a_*, and the critical threshold for nonuniversal gun ownership is $\left(b_{g}-c_{g}\right)/\left(\delta_{s}-\delta_{e}\right)$, which is greater than the threshold $\left(b_{g}-c_{g}\right)/\left(2\delta_{s}\right)$ above which social optimum is nonuniversal gun ownership.

Regardless of the sign *b_g_* − *c_g_*, for both scenarios, the population ends up overarming. In other words, what is best for individuals (x*) is at odds with what is best for society as a whole (*x^s^*). Such misalignment between individual self-interest and population interest leads to the social dilemma of overarming.

### Population structure: Lattice populations

Real populations are not generally well-mixed; instead, they have “spatial structure,” often represented as a network. We begin with a set of individuals situated on a square lattice with periodic boundary conditions (edges can represent social connections or actual spatial loci, so in this case, one can think of the connections as neighborhood arrangements). In addition to agent-based simulations, for the spatial dynamics of overarming, the method of pair approximation can be used to obtain analytical predictions. Instead of considering the frequency of strategies as in well-mixed populations, pair approximation traces the changes of strategy pairs (*61*). Let $p_{s,s^{\prime }}$ denote the probability of finding an individual playing strategy *s* accompanied by a neighbor playing $s^{\prime }$. Here, $s,s^{\prime }$ are either arming (by owning a gun), *A*, or not arming (not in possession of any guns), *B*. For the purpose of illustration, let us consider individuals arranged on a square lattice with the von Neumann neighborhood. Accordingly, the time evolution of different pairs can be determined by a system of ordinary differential equations

$$\begin{array}{c} {\dot{p}}_{A,A}=\sum_{x,y,z}\left[n_{c}\left(x,y,z\right)+1\right]p_{B,x}p_{B,y}p_{B,z}\sum_{u,v,w}p_{A,u}p_{A,v}p_{A,w}f\left[P_{A}\left(u,v,w\right)-P_{B}\left(x,y,z\right)\right]\\ -\sum_{x,y,z}n_{c}\left(x,y,z\right)p_{A,x}p_{A,y}p_{A,z}\sum_{u,v,w}p_{B,u}p_{B,v}p_{B,w}f\left[P_{B}\left(u,v,w\right)-P_{A}\left(x,y,z\right)\right],\\ {\dot{p}}_{A,B}=\sum_{x,y,z}\left[1-n_{c}\left(x,y,z\right)\right]p_{B,x}p_{B,y}p_{B,z}\sum_{u,v,w}p_{A,u}p_{A,v}p_{A,w}f\left[P_{A}\left(u,v,w\right)-P_{B}\left(x,y,z\right)\right]\\ -\sum_{x,y,z}\left[2-n_{c}\left(x,y,z\right)\right]p_{A,x}p_{A,y}p_{A,z}\sum_{u,v,w}p_{B,u}p_{B,v}p_{B,w}f\left[P_{B}\left(u,v,w\right)-P_{A}\left(x,y,z\right)\right] \end{array}$$ (5)

where *n_c_*(*x*, *y*, *z*) denotes the number of armed individuals among *x*, *y*, *z*, and the transition probability *f*(⸱⸱⸱) denotes the probability of an individual *i* adopting one of her neighbors *j*’s strategy. In this work, we use the Fermi function

$$f\left(\Delta P\right)=\frac{1}{1+\text{exp}\left[-\left(P_{j}-P_{i}\right)/K\right]}$$ (6)

The parameter *K* quantifies the “temperature” of selection and can be interpreted as the level of rationality in our cultural evolution context. K→0 means perfect rationality, whereas K≫1 is often called the limit of weak selection.

For simplicity, the above equations omit the common factor $2p_{A,B}/\left(p_{A}^{3}\cdot p_{B}^{3}\right)$ (and also the degree of the lattice *k*), which corresponds to a nonlinear transformation of the timescale but leaves the equilibrium unaffected. Under the symmetry condition $p_{A,B}=p_{B,A}$ and the normalization condition $p_{A,A}+p_{A,B}+p_{B,A}+p_{B,B}=1$, the above equations are closed and can be numerically solved (integrated) to get equilibrium values ${\tilde{p}}_{A,A}$ and ${\tilde{p}}_{A,B}$. These equilibrium values in turn lead to an approximation of the equilibrium frequency of armed individuals (the percentage of gun ownership) ${\tilde{p}}_{A}={\tilde{p}}_{A,A}+{\tilde{p}}_{A,B}$. The complement of ${\tilde{p}}_{A}$ gives the equilibrium frequency of unarmed individuals, ${\tilde{p}}_{B}=1-{\tilde{p}}_{A}$.

### Real social networks

Spatial lattice populations serve as an initial means to account for local clustering in social networks. However, to more accurately represent the real world’s degree heterogeneity, a common feature in social interactions, we must consider real social networks and specifically how their network degree heterogeneity affects individuals’ choices regarding gun acquisition. These real networks, including a gang network from Montreal, Canada (*62*), a village network from Honduras (*65*), and a college campus network from the United States (*63*, *64*), are constructed from sociomatrices obtained through interviews or the name generator technique. Within these networks, individuals occupy varying positions, with some being more central than others. We study the evolutionary dynamics of arming choices on both real and synthetic heterogeneous networks. The synthetic degree-heterogeneous network (Fig. 3A) is based on the scale-free network model (*68*). The simulation process on networks closely resembles that of the lattice populations mentioned earlier. Our simulations use only the anonymized social tie information from these real networks, as already published in (*62*–*65*). Evidently, the underlying network structure significantly influences interactions and the acquisition of firearms. Certain networks display increased vulnerability to excessive overarming. This highlights the critical role of social networks in the spread of gun acquisitions and therefore development of effective gun control strategies.

### Model extensions

In the Supplementary Materials, we also present detailed analyses of model extensions, including the impact of potential interventions aimed at perturbing the underlying social network to mitigate overarming, the dynamics of gun interactions in group settings, and the coevolution of gun acquisition and risk perception. Regarding modeling assumptions related to the negative-sum payoff structure and heterogeneous behavioral types of gun owners, we further consider two extensions: (i) breaking the zero-sum assumption in *AB* encounters by introducing a discounting parameter α, and (ii) incorporating a third type of “fair-use” gun owners whose decision to use a gun in a confrontation depends on whether the other party is armed and also uses a gun.

## Supplementary Materials

### This PDF file includes:

Supplementary Text Tables S1 to S3 Figs. S1 to S11 References
