# Supplementary material for: Bivalent impact of social networks on overarming: Insights on the alignment between social and individual interests
Source: Sci Adv. 2026 Jun 3;12(23):eaed3904. doi: 10.1126/sciadv.aed3904 (PMC13232554; doi:10.1126/sciadv.aed3904)
Supplement: Supplementary file 1 — Supplementary Text Tables S1 to S3 Figs. S1 to S11 References [file sciadv.aed3904_sm.pdf]

Science Advances
AAAS

Supplementary Materials for

Bivalent impact of social networks on overarming: Insights on the alignment between social and individual interests

Feng Fu *et al.*

Corresponding author: Feng Fu, feng.fu@dartmouth.edu

Sci. Adv. **12**, eaed3904 (2026)
DOI: 10.1126/sciadv.aed3904

This PDF file includes:

Supplementary Text
Tables S1 to S3
Figs. S1 to S11
References

Supplementary Text

Quantifying network structural effect on arming choices

As opposed to well-mixed populations, the presence of spatial population structure, and social network structure in general, renders a double-edged sword effect on individual decisions regarding gun ownership. That is, the critical p_a above which gun acquisitions are non-zero in structured populations is greater than that in well-mixed populations. Moreover, overarming is greatly exacerbated in the former than in the latter for larger p_a values. To shed analytical insight into this nontrivial phenomenon, we focus on the local clustering arising spatial populations. Another important characteristic of network populations is degree heterogeneity, which we will analyze separately. Here, we restrict our analysis to costly individual gun ownership, $b_g - c_g < 0$; other scenarios can be analyzed analogously, as detailed in the model extensions below.

Using the method of pair approximation, we are able to calculate the local clustering (or assortment) analytically in the limiting case of weak selection $K \gg 1$. In this case, the local densities, for example, $q_{A|A}$ (the conditional probability of an A individual finding another A in its direct neighborhood), reach equilibrium values much faster than the global density of A, denote by x. Using the technique of timescale separation and solving the dynamics along slow and fast manifolds respectively, we obtain:

$$q_{A|A} = x + \frac{1}{k-1}(1-x), \tag{S1}$$

$$q_{B|B} = 1 - \frac{k-2}{k-1}x. \tag{S2}$$

Considering the strategy updating along the boundary between A and B individuals, we focus on a randomly chosen AB pair in the population. For this A individual, its expected payoff $g_1(x)$ is

$$g_1(x) = b_g - c_g + \frac{p_a}{k}\left[\delta_e + (k-1)((-1)q_{A|A} + \delta_e q_{A|A})\right]. \tag{S3}$$

Similarly, the expected payoff for the B individual is given by

$$g_0(x) = \frac{p_a}{k}\left[-\delta_e + (k-1)((-\delta_e)q_{A|B} - \delta_n q_{B|B})\right]. \tag{S4}$$

In order for A individuals to emerge in the population, we need the condition $g_1(0) > g_0(0)$. Therefore we obtain the threshold p_a required for A strategy to emerge in the population when

initially rare:

$$p_a > \frac{k(c_g - b_g)}{(k-1)\delta_n + k\delta_e - 1}. \tag{S5}$$

By solving $g_1(x) = g_0(x)$, we obtain the equilibrium fraction of gun acquisition in spatial populations under the weak selection limit:

$$x^* = \begin{cases} 0, & p_a \leq \frac{k(c_g-b_g)}{(k-1)\delta_n+k\delta_e-1} \\ \frac{-p_a(\delta_n+\delta_s)+k[b_g-c_g+p_a(\delta_n+\delta_e)]}{(k-2)p_a(\delta_n+\delta_s)}, & p_a > \frac{k(c_g-b_g)}{(k-1)\delta_n+k\delta_e-1}. \end{cases} \tag{S6}$$

Payoff structure condition for double-edged sword effect: the fear of being confronted with gun owners As pointed out before, both the payoff value δ_e and the relative rate of provocation p_a impact the severity of the social dilemma of overarming. Because of clustering (kind-meets-kind) in spatial populations, the effective social payoff for gun interactions (the effective δ_e value) is shifted as in the following:

$$\begin{pmatrix} -\delta_s & \delta_e \\ -\delta_e & -\delta_n \end{pmatrix} \longrightarrow \begin{pmatrix} -\delta_s & \delta_e + \frac{2\delta_e-\delta_s+\delta_n}{k-2} \\ -\delta_e - \frac{2\delta_e-\delta_s+\delta_n}{k-2} & -\delta_n \end{pmatrix}$$

Clearly, the payoff structure condition for spatial structure to aggravate the social dilemma of overarming possibly at all as opposed to well-mixed scenarios if and only if $\delta_e > \frac{1}{2}(\delta_s - \delta_n)$.

For the same reason, the individual (nonsocial) payoff matrix for arming decisions is

$$\begin{pmatrix} b_g - c_g & b_g - c_g \\ 0 & 0 \end{pmatrix} \longrightarrow \begin{pmatrix} b_g - c_g & b_g - c_g + \frac{2(b_g-c_g)}{k-2} \\ -\frac{2(b_g-c_g)}{k-2} & 0. \end{pmatrix}$$

Furthermore, in order to allow the crossover of the two equilibrium curves from well-mixed and spatial populations respectively, we obtain the overall payoff structure condition:

$$\delta_e > c_g - b_g + \frac{1}{2}(\delta_s - \delta_n). \tag{S7}$$

More generally, in agent-based simulations, the critical values δ_e will be smaller than this theoretical prediction for two reasons. First, this prediction is obtained under the limit of weak selection and non-weak selection will lead to higher equilibrium fraction of A individuals. Second, the method of pair approximation underestimates the population equilibrium of A individuals.

Under this condition, spatial structure exhibits a double-edged sword effect on gun acquisitions, depending on the relative provocation rate p_a. The critical value p_a^* is given by

$$p_a^* = \frac{2(c_g - b_g)}{2\delta_e - (\delta_s - \delta_n)} \leq 1. \tag{S8}$$

For $p_a < p_a^*$, spatial structure actually inhibits gun acquisitions as compared to well-mixed populations, whereas for $p_a > p_a^*$, spatial structure promotes gun acquisitions, thereby leading to greater extent of overarming.

Another interesting condition for spatial structure to completely inhibit A is

$$\delta_e < c_g - b_g + \frac{\delta_s - (k-1)\delta_n}{k}. \tag{S9}$$

If this condition holds, spatial structure is always a suppressor of gun acquisitions. There is no way for gun acquisitions to unfold in spatial populations. This theoretical result has significant real-world implications for gun control.

Understanding the impact of network degree heterogeneity: dynamics on star graphs under strong selection Although it is possible to study the invasion and fixation dynamics of individual arming choices on graphs/networks using an explicit Markov chain approach with absorbing states, the numerical calculation becomes impractical especially for large network sizes. Nevertheless, to understand the impact of network heterogeneity on overarming, we can instead focus on graphs that are simple yet still capture the essence of the topological features including degree heterogeneity and clustering.

Recall that the gun acquisition dynamics on the gang and campus networks exhibit qualitatively different behavior than the village network: that is, there exists a sharp transition from zero to complete gun acquisitions as the relative provocation rate p_a increases. The gang and campus networks resemble "star-like" graphs. Thus, let us simulate the same game dynamics of gun acquisitions on star graphs (zero clustering), wheel graphs (leaf nodes connected via a cycle, leading to some level of clustering), double stars, and double wheels (see Fig. S3). We find that the network heterogeneity in these simple examples greatly exacerbates overarming and moreover, the presence of local clustering (wheels and double wheels, as we have explained the analytical insights for degree regular graphs such as spatial populations) can inhibit the emergence of gun

acquisitions for small p_a, leading to similar results we have observed in the degree-heterogeneous model networks. The real gang and campus networks have "star-like" motifs that share similarity with wheels and double wheels, whereas the model degree-heterogeneous network and the real village network share topological similarity and therefore they lead to qualitatively same dynamics of gun acquisition.

The star graphs allow mathematical tractability and provide further analytical intuitions for understanding our agent-based simulation results on social networks. In our simulations, we use synchronous updating where every individual updates their arming choices based on the Fermi function at each time step (also known as the pairwise comparison rule (*74*)). In general, synchronous updating leads to qualitatively the same results as asynchronous updating in which each individual updates their strategy in a random sequential manner. However, noticeable difference can arise under certain conditions such as strong selection. Under this limit, individuals only adopts the strategy of a randomly chosen neighbor only if the neighbor has a higher payoff than themselves.

Specifically, we consider the way in which gun acquisitions (denoted by A) spread in a star network that has zero gun acquisitions initially (denoted by B). Despite being much more complicated, the case of wheels can be analyzed analogously and leads to additional insights about clustering besides heterogeneity. The fixation probability of a single A, starting on the periphery or the center, can be calculated in closed form for star graphs. On the other hand, the conditional fixation time can be exponential, making it impossible to witness fixation in simulations for non-weak selection. Furthermore, we show that the stochastic dynamics become semi-deterministic under the limit of strong selection ($K \to 0$), and that reaching fixation is not always possible. In fact, under certain conditions the star network can enter into one of the two possible configurations that the system alternates between: the center probabilistically alternates between A and B while all the leaf nodes are "frozen" in their states and thus maintain a fixed proportion of A and B.

Consider a star graph of size $n + 1$ (the number of leaf nodes is n) and asynchronous updating of strategies in the limit of strong selection. As noted above, synchronous updating leads to instantaneous fixation as soon as the center adopts A and has a higher payoff than leaf nodes. For this reason, we focus on asynchronous updating, which leads to more interesting dynamic behavior.

If an A appears on one of the leaf nodes, this A first needs to take over the center B in order to spread to other leaf nodes. The expected payoff of the center B against 1 A and $(n - 1)$ B's in its

neighborhood is

$$g_0^H(1) = p_a(-(n-1)\delta_n - \delta_e)/n, \tag{S10}$$

while the expected payoff of the leaf node A against a center B is

$$g_1^L(B) = b_g - c_g + p_a(\delta_e), \tag{S11}$$

Thus it requires $g_1^L(B) > g_0^H(1)$, which leads to a condition formally similar to well-mixed populations for large n:

$$p_a > \frac{n(c_g - b_g)}{(n+1)\delta_e + (n-1)\delta_n}. \tag{S12}$$

If an A appears on the center initially, its expected payoff, $b_g - c_g + p_a\delta_e$, must be greater than those leaf nodes B's, $-p_a\delta_e$, thereby leading to the condition

$$p_a > \frac{c_g - b_g}{2\delta_e}. \tag{S13}$$

Because of the nature of negative interactions, the center A's payoff is strictly decreasing with the number of A's, denoted by i, in its neighborhood. Meanwhile, given the center is an A, the expected payoff of a periphery B, $g_0^L(A) = -p_a\delta_e$, is greater than that of a periphery A , $g_1^L(A) = b_g - c_g - p_a\delta_s$, because $b_g - c_g < 0$ and $\delta_s > \delta_e$. Thus, regardless of whether the star graph starts with a single A at its center or at one of the periphery nodes, the center must adopt and remain as A in order for A's to propagate through the star graph. However, such propagation of A's in the star network will stall after the number of A's i reaches a critical threshold i^* at which the center A's expected payoff $g_1^H(i) = b_g - c_g + p_a[-i\delta_s + (n-i)\delta_e]/n$ is less than any periphery B's, $g_0^L(A) = -p_a\delta_e$. This condition leads to

$$i > \frac{n(2p_a\delta_e + b_g - c_g)}{p_a(\delta_s + \delta_e)} = i_c^A \tag{S14}$$

When the number of A reaches $\lfloor i_c^A \rfloor + 1$, the center A will turn into a B with probability $[n - (\lfloor i_c^A \rfloor + 1)]/n$ since it has smaller expected payoff than any periphery B. Once the center turns into B, it needs to be taken over by A again, or else the number of A's will drop in the star network as the center B can take over periphery A's as long as it has higher payoff than A. This could lead to an amplifying effect on the demise of A's, since a center B's expected payoff strictly increases as the number of periphery A's decreases. As such, we obtain another critical threshold i_c^B for the number

of A's in the periphery:

$$g_1^L(B) = b_g - c_g + p_a\delta_e > g_0^H(i) = p_a(-i\delta_e - (n-i)\delta_n)/n \tag{S15}$$

We have

$$i > \frac{n[c_g - b_g - p_a(\delta_e + \delta_n)]}{p_a(\delta_e - \delta_n)} = i_c^B \tag{S16}$$

When both conditions are satisfied, the states of periphery nodes are frozen (the number of periphery A's is given by $\lfloor i_c^A \rfloor + 1$) whereas the center node alternates between A and B probabilistically (see a simulation result in Fig. S4).

Figure S5 depicts the regions of model parameters in which the final state the system will approach, depending on whether the center is started with A or B. If the center of the star graph starts with A, the most interesting region is that the system can initially increase with A due to propagation of A from the center to periphery nodes but once the number of A's in the leaf nodes exceeds a critical threshold that the center A has a smaller payoff than a periphery B, it quickly leads to the demise of all A's. If the center of the star graph starts with B, the most interesting region is the bistability region: only when the initial number of A's in the leaf nodes exceeds a critical threshold, A's are able to sustain themselves in the population. In both cases, the population either has zero gun acquisitions or end up with a mix of A and B where the center alternates between A and B while leaf nodes are fixed in their proportion of A and B. Note that the equilibrium fraction of gun acquisitions on star graphs is greater than in well-mixed populations (Fig. S4).

Community gathering: more guns, more gun-related incidences

In certain settings, interactions extend beyond one-on-one engagements and involve groups of individuals. These group gatherings commonly occur in locations such as schools, shopping centers, churches, and public demonstrations or protests. With the rise in gun ownership, the likelihood of firearms being present at these events also increases. In this analysis, we conduct a straightforward calculation that illustrates how the inevitability of human error leads to a rise in gun-related incidents as the presence of firearms grows. Although our calculations employ a simplified probabilistic approach, they provide valuable insights that challenge the misconception that "more guns lead to a safer community."

The risk of everyday gun-related incidence for gathering size G Let ϵ denote the error probability that an armed individual causes a gun-related incidence, and let p denote the probability that an individual participating in community gatherings is armed. Then the expected loss for a gathering of G individuals, expressed as the occurrence likelihood of at least one error:

$$\sum_j \binom{G}{j} p^j (1-p)^{G-j} (1-(1-\epsilon)^j) \tag{S17}$$

$$= 1 - ((1-\epsilon)p + (1-p))^G \tag{S18}$$

$$= 1 - (1 - p\epsilon)^G \tag{S19}$$

Therefore, the risk of gun-related incidence increases monotonically with the arming probability p and the error probability ϵ, and for large gathering sizes $G \gg 1$, this risk becomes almost certain except for zero gun possessions ($p = 0$) or carrying free of human errors ($\epsilon = 0$).

Narrow margin to enable beneficial outcomes for "good guys with guns" scenario Suppose some fraction c of the population are criminals, and the remaining fraction $1 - c$ are citizens. We assume that a fraction p of the population is armed all the time. Any armed person makes harmful mistakes (errors) with probability ϵ (per day carry).

Let G be the size of a community gathering, and let us assume a random assortment of citizens so that pG are armed and $(1 - p)G$ are unarmed.

The likelihood A_0 of an everyday scenario of gatherings with citizens only is

$$A_0 = \binom{G}{0} c^0 (1-c)^G = (1-c)^G.$$

The likelihood A_1 of a "threat" scenario of gatherings of citizens but mixed with a single criminal is

$$A_1 = \binom{G}{1} c^1 (1-c)^{G-1} = Gc(1-c)^{G-1}.$$

The expected loss for everyday scenario among armed citizens (the cost of carrying firearms) is $C(G, p, \epsilon) = 1 - (1 - p\epsilon)^G$.

The expected probability of successful deterrence (the benefit of carrying firearms) in a threat scenario which occurs in relative frequency $K_0 = A_1/A_0$ to everyday scenarios:

$$B(G, p, \epsilon) = K_0 \cdot \sum_j \binom{G-1}{j} p^j (1-p)^{G-1-j} (1-\epsilon^j) = K_0(1 - (p\epsilon + 1 - p)^{G-1})$$

which monotonically decreases with ϵ. For a wide range of parameters, there exists only a very narrow margin of human error ϵ to ensure net beneficial outcomes for "good guys with guns" scenario, i.e., $B(G, p, \epsilon) > C(G, p, \epsilon)$.

Model extensions

Impact of small, targeted network changes on gun ownership As mentioned above, social network structure impacts gun acquisition. Certain network structures, like star graphs, strongly amplify gun acquisition, whereas others, like wheels, have a lesser impact. While our approach can be used to study any given network topology and assess its impact on gun acquisition as an amplifier vs suppressor, we focus specifically on how small changes in the network can have meaningful effects. This question has practical implications for social interventions aimed at reducing overarming.

Without loss of generality, we demonstrate the impact of adding a single edge between a pair of nodes with the largest path length in the real campus and village networks, respectively (as shown in Fig. 3 of the main text; also Fig. S6 and S7). In the campus network, the added link in Fig. S6B the previously two leaf nodes are now joined by the new added link, forming a large cycle. Despite this small change, the impact on gun acquisition is pronounced: it increases the threshold of p_a, above which the population abruptly transitions from zero to full acquisition especially with lower gun acquisition rates most clearly seen in the transition. The intuition is that joining two distant leaf nodes, each from a local star motif, weakens the relative advantages of the local hubs they are connected to due to their increased connectivity (also see Fig. S2) and therefore leads to a lower inclination to gun acquisition. For this reason, we also see similar difference between wheel and star graphs (Fig. S3). Furthermore, we confirm a comparable yet noticeable impact of small, targeted network changes in the real village network, as shown in Fig. S7, albeit less pronounced compared to Fig. S6 (since the latter network is not as strong an amplifier of gun acquisition as the former, see Fig. 3 in the main text). Taken together, these results lend support for optimizing social network interventions via "graph surgery," which only needs small changes in the network but can yield a positive impact as desired.

Group interactions beyond pairwise encounters It is of interest to study how negative group interactions where each individual confronts against one another impact gun ownership rates. Assume the frequency of A individuals in a population is x and the frequency of B individuals is $1 - x$. We consider unbiased group formation: a group of size m is formed by binomial sampling from the perspective of a focal individual. Therefore, the probability of having k A's among the $m - 1$ group members excluding the focal individual is given by:

$$\binom{m-1}{k} x^k (1-x)^{m-1-k}.$$

For a group size m, the payoffs associated with A (armed) and B (disarmed) depend in the number of A's, denoted by k, in the group. Specifically, we assume the expected average escalation payoff for an A individual due to a shootout linearly depend on the fraction of A as $-\delta_s(k-1)/m$ while the average intimation payoff for A obtained from B individuals is shared with all A's, δ_e/k for $k \geq 1$. On the other hand, a B individual in a group having k A's has a concession cost as δ_e only $k \geq 1$; otherwise it is zero. In addition, the average payoff for a B individuals in unarmed confrontations is $-\delta_n * (m-k-1)/m$. This means for an individual, A or B, centered group consisting of k many other A's:

$$g_{1,k} = -\delta_s k/(m-1) + \delta_e/(k+1) * (m-1-k)/(m-1),$$

$$g_{0,k} = -\operatorname{sign}(k)\delta_e/(m-1) - \delta_n * (m-k-1)/(m-1),$$

Averaging all possible group compositions, we obtain the average payoff for an A individual

$$g_1(x) = b_g - c_g + p_a \sum_{k=0}^{m-1} \binom{m-1}{k} x^k (1-x)^{m-1-k} g_{1,k} = b_g - c_g + p_a \frac{-\delta_s(m-1)x^2 + \delta_e[(1-x)-(1-x)^m]}{(m-1)x}$$

and the average payoff for a B individual

$$g_0(x) = p_a \sum_{k=0}^{m-1} \binom{m-1}{k} x^k (1-x)^{m-1-k} g_{0,k} = p_a[-\delta_e \frac{1-x-(1-x)^m}{(m-1)(1-x)} - \delta_n(1-x)]$$

The ESS $x^* \in (0,1)$ can be solved numerically by letting $g_1(x) = g_0(x)$. As shown in Fig. S8, x^* exhibits similar transition from zero to non-zero acquisition once the provocation rate p_a exceeds a critical threshold.

In particular, for $m = 2$, the model reverts to our original model with pairwise encounters:

$$\begin{cases} g_1(x) & = b_g - c_g + p_a(-\delta_s x + \delta_e(1-x)), \\ g_0(x) & = p_a(-\delta_e x - \delta_n(1-x)). \end{cases}$$

For $m > 2$, the model shows the impact of group size on equilibrium gun ownership rate. Because of the antagonistic nature of gun interactions, gun acquisition rate is lower in larger groups (Fig. S8). The presence of more guns does not render higher advantage over unarmed individuals but instead they neutralize at each other and diminish the potential advantage in confrontations.

Interestingly, the critical p_a above which non-zero gun acquisition arises remain the same, regardless of the group size m, given by letting $g_1(x) = g_0(x)$ and solving for p_a

$$p_a^* = \lim_{x \to 0} \frac{(b_g - c_g)(m-1)x(x-1)}{\delta_e(1 - x - (1-x)^m) + \delta_n(m-1)x + (2\delta_n + \delta_s)(1-m)x^2 + (m-1)(\delta_s + \delta_n)x^3} \tag{S20}$$

$$= \frac{c_g - b_g}{\delta_e + \delta_n}.$$

This independence of p_a^* on m is also confirmed in Fig. S8.

Threat perception co-evolving with gun acquisitions In our base model, we use the provocation rate p_a to prescribe the extent of social environment deterioration and how it impacts individuals' decisions to own guns. More realistically, this parameter p_a can be interdependent with the population-level gun ownership rate x at any given time and thus dynamically coevolve with it. Therefore, we consider an extended model where $p_a(t)$ represents individuals' threat perception – an individual's belief about the likelihood of encountering confrontations involving guns – as follows:

$$\dot{x} = x(1-x)(b_g - c_g + p_a(\delta_e + \delta_n - (\delta_n + \delta_s)x)), \tag{S21}$$

$$\dot{p_a} = p_a(1-p_a)(\theta x - (1-x)). \tag{S22}$$

The parameter $\theta > 0$ represents how strongly individuals perceive increased threat due to others' gun acquisitions x which is also counteracted by the fraction of the population choosing not to arm themselves, $1 - x$. Thus, the term $\theta x - (1-x)$ regulates how threat perception coevolves with gun acquisitions x.

On the other hand, the perceived threat p_a impacts individuals' deliberations of payoffs, we have $g_1(x) = b_g - c_g + p_a(-\delta_n x + \delta_e(1-x))$, and $g_0(x) = p_a(-\delta_e x - \delta_n(1-x))$. Substituting these payoffs into the replicator equation, we obtain the system of ordinary differential equations above.

Table S1 lists all possible equilibria (fixed points) of the co-evolving system (x^*, p_a^*). The eigenvalues of the Jacobian matrix at each fixed point provide insights into their stability. Aside from the boundary equilibria, we are mainly interested in the condition for the existence of an interior fixed point for $0 < x^* < 1$ and $0 < p_a^* < 1$, requiring

$$0 < \frac{1}{1+\theta} < 1, \tag{S23}$$

$$0 < \frac{(c_g - b_g)(1+\theta)}{\delta_e - \delta_s + \theta(\delta_e + \delta_n)} < 1. \tag{S24}$$

The first inequality is satisfied for any $\theta > 0$. Therefore, for the second inequality to hold, the payoff structure must satisfy certain conditions as given above. Whether for the negative solo payoff of owning a gun ($b_g - c_g < 0$, which is the focus of the main text) or the opposite case ($b_g - c_g > 0$), this interior fixed point can exist.

The stability of these fixed points is worth noting. All boundary equilibria are either a stable node (when both eigenvalues of the Jacobian matrix are negative) or an unstable node (when both eigenvalues are positive) or a saddle point (when the eigenvalues have opposite signs). Interestingly, the only admissible interior fixed point $(\frac{1}{1+\theta}, \frac{(c_g-b_g)(1+\theta)}{\delta_e-\delta_s+\theta(\delta_e+\delta_n)})$, when it exits, is either an unstable saddle point or a stable spiral. To be specific, the trace and determinant of the Jacobian matrix J associated with this fixed point are given by:

$$\mathrm{Tr}(J) = \frac{(b_g - c_g)(\delta_n + \delta_s)\theta}{(1+\theta)(\delta_e - \delta_s + (\delta_e + \delta_n)\theta)}, \tag{S25}$$

$$\mathrm{Det}(J) = \frac{(b_g - c_g)(b_g - c_g + \delta_e - \delta_s + (b_g - c_g + \delta_e + \delta_n)\theta)\theta}{(\delta_e - \delta_s + (\delta_e + \delta_n)\theta)(1+\theta)}. \tag{S26}$$

Typically, confrontations involving guns are far more detrimental than those without, meaning $\delta_s \gg \delta_e, \delta_n$. As such, $b_g > c_g$ for the solo payoff of owning a gun needs to hold to ensure $\mathrm{Tr}(J) < 0$. Indeed, this condition is particularly relevant in the context of American gun culture. Our modeling analysis suggests that gun acquisition and risk perception together can manifest oscillatory behavior, even without accounting for external disruptions such as social unrest.

Therefore, when $(\mathrm{Tr}(J))^2 - 4\,\mathrm{Det}(J) < 0$ the fixed point is a stable spiral, indicating the system can exhibit damped oscillatory behavior. When $\mathrm{Det}(J) < 0$ and $(\mathrm{Tr}(J))^2 - 4\,\mathrm{Det}(J) > 0$, the fixed point is a saddle point. However, it is impossible for $\mathrm{Det}(J) > 0$ (the product of the two eigenvalues being positive) while $\mathrm{Tr}(J) < 0$ (their sum being negative) under the payoff structure requiring $\delta_s \gg \delta_e, \delta_n$ and $b_g > c_g$.

In the main text, Fig. 5 presents the system dynamics through streamplots in the phase plane of (x, p_a), along with the admissible boundary and interior equilibria, by comparing scenarios where $b_g < c_g$ (Fig. 5a and 5b) to those where $b_g > c_g$ (Fig. 5c and 5d). In line with the main case discussed in the main text ($b_g < c_g$, where the solo payoff for owning a gun is negative), a small feedback effect of the corresponding perceived threat in response to others' gun acquisition (small θ) results in a single stable equilibrium at $(0, 0)$. This suggests that the population ultimately converges to complete disarmament, leading to zero perceived risk. In contrast, as the feedback effect increases significantly (large θ), an interior equilibrium $(\frac{1}{1+\theta}, \frac{(c_g-b_g)(1+\theta)}{\delta_e-\delta_s+\theta(\delta_e+\delta_n)})$ emerges, which is a saddle point. In this regime, the system exhibits bistable dynamics: depending on the initial conditions, the population can converge to either the disarmed state $(0, 0)$ or the state of high gun acquisition rate $\frac{b_g-c_g+\delta_e+\delta_n}{\delta_n+\delta_s}, 1)$. The respective basins of attraction for these two stable equilibria can be seen in Fig. 5b.

In the complementary case where $b_g > c_g$ (individual payoff for owning a gun is positive), the population shows a natural preference for gun ownership in the first place. This scenario may align with cultural contexts such as American gun culture, where gun ownership is often, though not universally, perceived positively when social payoffs are not considered. In this case with $\delta_s \gg \delta_e, \delta_n$, the same interior point $(\frac{1}{1+\theta}, \frac{(c_g-b_g)(1+\theta)}{\delta_e-\delta_s+\theta(\delta_e+\delta_n)})$ becomes a stable spiral under certain payoff structure conditions. Notably, the population show oscillatory dynamics in gun acquisition and risk perception. As gun acquisition rises in response to perceived risk (which is elevated by others' gun acquisition), the exceedingly high prevalence of guns subsequently diminishes the perceived relative advantage of ownership. This feedback loop can further lead to lower gun acquisition and reduced risk perception, creating oscillations even in the absence of external disturbances such as social unrest. These dynamics highlight the antagonistic nature of gun interactions and the oscillating "tragedy of the commons" driven by self-fulfilling cycles of gun acquisition and threat perception.

Finally increasing the cost of concession δ_e to a level comparable to δ_s causes the interior

Table S1: List of all possible fixed points and the eigenvalues of the Jacobian matrix J for the corresponding fixed points. We use ** only in cases where the closed-form expressions for eigenvalues $\lambda_{1,2}$ are too lengthy to include. The eigenvalues for a 2×2 Jacobian matrix J are given by $\lambda_{1,2} = \frac{1}{2}(\mathrm{Tr}(J) \pm \sqrt{(\mathrm{Tr}(J))^2 - 4\,\mathrm{Det}(J)})$.

**Fixed points** (x^*, p_a^*)	**Eigenvalues**
$(0, 0)$	$-1, b_g - c_g$
$(1, 0)$	$-b_g + c_g, \theta$
$(0, 1)$	$1, b_g - c_g + \delta_e + \delta_n$
$(1, 1)$	$-b_g + c_g - \delta_e + \delta_s, -\theta$
$(\frac{b_g - c_g + \delta_e + \delta_n}{\delta_n + \delta_s}, 1)$	$\frac{(b_g - c_g + \delta_e + \delta_n)(b_g - c_g + \delta_e - \delta_s)}{\delta_n + \delta_s}, -\frac{b_g - c_g + \delta_e - \delta_s + \theta(b_g - c_g + \delta_e + \delta_n)}{\delta_n + \delta_s}$
$(\frac{1}{1+\theta}, \frac{(c_g - b_g)(1+\theta)}{\delta_e - \delta_s + \theta(\delta_e + \delta_n)})$	**

equilibrium to disappear while the boundary equilibrium becomes stable. In this example shown in Fig. 5d, the population ends up with heightened risk perception with high gun acquisition. Varying δ_e has a similar impact as in the base model studied in the main text (Fig. 1b): the fear of being disadvantaged can drive a self-reinforcing cycle between overarming and persistent perceive risks.

Model-based insights into gun purchases in the United States since 2000

The extended coevolutionary model of gun acquisition and threat perception incorporates subtle psychological dynamics in a feedback manner: perceived provocation can be influenced by others' gun acquisition, which, in turn, can trigger further threat response, such as purchasing more guns, due to elevated perceived risks. A theoretical model of this sort, while not taking into account external social and political events, provides plausible mechanistic insights into the self-reinforcing cycles of self-serving gun acquisition and risk perception, particularly in the context of American gun culture.

To this end, as a reasonable and practical consideration, we use annual gun sales in the United States since 2000 as a proxy for threat responses driven by perceived risks. This approach does not account for the seasonality of gun purchases, which exhibit cyclic patterns throughout the year. However, it still effectively captures changes in perceived risk and the overall trend from year to year.

Our data source is retrieved from urlhttps://www.thetrace.org/2020/08/gun-sales-estimates/ which is derived from FBI's National Instant Criminal Background Check System (NICS). Because the values of perceived risk p_a and gun acquisition x are restricted to $[0, 1]$ without loss of generality, we use the normalized original data (plotted as dots in Fig. S9). We then fit our model and obtain the best estimates for the model parameters using the simulated annealing method (Table S2). The inferred model parameters reveal a specific payoff structure satisfying $b_g > c_g$ and $\delta_s \gg \delta_e > \delta_n$. Given these estimated payoff values, the population exhibits oscillatory dynamics, as predicted by our theoretical analysis in above Section (Fig. 5c in the main text). The alignment between the data and the fitted model suggests that our extended model effectively captures the interplay between self-serving inclination to own a gun as a threat response and the dynamics of risk perception.

Despite the insights provided by our extended model, several limitations warrant discussion. First, the data on gun purchases, derived from background checks, is an inevitable underestimate of actual gun acquisition due to private sales, unreported transactions, and varying state regulations. This discrepancy highlights the need for more comprehensive data to fully capture gun acquisition trends.

Second, the model does not account for local variations in gun culture or differences between states, which are significant factors in shaping attitudes toward gun acquisition and perceived risk. For instance, states with strong traditions of hunting or permissive gun laws may exhibit dynamics distinct from those with stricter regulations or differing cultural attitudes.

Third, the model does not account for major external events, such as the 2008 financial crisis and the social unrest during the COVID-19 pandemic, both of which likely influenced gun sales and public perceptions of risk. Incorporating such contextual factors could further enhance the model's explanatory and predictive power, particularly with regard to the goodness of fit during the 2020s. Nevertheless, the fact that the model captures the overall trend indicates it captures the essence of

the interplay between gun acquisition and risk perception.

It is also important to note that this is a population-level model, which, while not capturing individual-level variability or decision-making, is highly useful in providing a broad understanding of the feedback dynamics at play. Future extensions of the model could incorporate additional factors such as social norms, legislative changes, media influence, and broader social behavioral dynamics of gun acquisition. Moreover, longitudinal studies examining attitudes and behaviors related to guns would be invaluable for refining and validating such models. These empirical studies could provide more granular data, enabling a deeper and more realistic exploration of gun acquisition and risk perception at both individual and community levels.

Table S2: Model parameter estimations based on real gun purchase data from 2000 to 2023. We present the best-fitted values of the model parameters relative to the cost of a shootout, δ_s, which is fixed at one without loss of generality.

Model parameters	Best estimates	95% confidence intervals
b_g	0.8281	$[0.3038, 0.9952]$
c_g	0.6417	$[0.1182, 0.8080]$
δ_e	0.0015	$[0.0010, 0.1000]$
δ_n	0.0012	$[0.0010, 0.0027]$
δ_s	1.0000	$[1.0000, 1.0000]$
θ	0.9037	$[0.7311, 1.0000]$

Empirical support for game-theoretic model parameters regarding gun ownership

In Table S3 below, we compile a list of U.S.-based studies and survey data that inform the operationalization of each parameter in our model, providing a brief summary of relevant qualitative and quantitative empirical findings. Doing so not only strengthens the empirical grounding of our theoretical work but also helps to conceive future empirical research.

To assess the plausibility of our theoretical assumptions and their relevance to real-world behavior, we fit our coevolutionary model to U.S. gun purchase data from 2000 to 2023. The best-fit model parameters (as given in Table S2 above) are qualitatively consistent with numbers observed in empirical studies and national survey reports (Table S3).

Specifically, the individual benefit of gun ownership ($b_g \approx 0.83$) exceeds its cost ($c_g \approx 0.64$), aligning with survey data indicating that most owners report a net sense of safety and utility from firearm possession. The social cost of shootouts is fixed at $\delta_s = 1$, allowing us to interpret the relative magnitudes of concession costs ($\delta_e \approx 0.0015$) and unarmed disputes ($\delta_n \approx 0.0012$). These low values reflect the common perception that the harm from yielding in an armed encounter or from verbal disputes is minimal relative to the potentially fatal consequences of gun violence. The inferred threat-sensitivity parameter ($\theta \approx 0.90$) indicates that individuals in our model strongly adjust their perceived risk based on others' gun acquisitions – a finding supported by psychological studies on the contagious fear of being outgunned. Together, these values ground the model in empirically consistent assumptions, while highlighting how population dynamics and subjective perceptions can coevolve to produce collective overarming.

While our model is not directly validated with individual-level behavioral data, this form of empirical parameter estimation provides a first step toward testing the model's predictions against real-world dynamics. As such, our approach supports hypothesis generation for future interdisciplinary empirical studies, including behavioral experiments, and longitudinal analysis of firearm acquisition patterns in structured populations.

Further extensions on negative-sum interactions and behavioral heterogeneity

Here we extend our base model by accounting for a negative-sum payoff structure in AB encounters and for heterogeneous behavioral types of gun owners. Specifically, we consider two extensions under alternative modeling assumptions: (1) breaking the zero-sum assumption in AB encounters by introducing a discounting parameter α, and (2) incorporating a third type of *fair-use* gun owners, whose decision to use a gun in a confrontation depends on whether the other party is armed and also uses a gun. Overall, our main conclusions regarding overarming remain qualitatively unchanged under these extensions.

Accounting for negative-sum AB encounters Our original base model assumes zero-sum interactions for AB pairs: the B player's loss (i.e., $-\delta_e$) equals the A player's gain (i.e., δ_e). However, it is not implausible that the total payoff of an AB pair can be negative-sum, as in AA and BB encounters, given the threat posed by guns and the overall antagonistic nature of such confrontations. To this end, we extend our base model by introducing a discounting parameter α for the payoff vector $(\alpha\delta_e, -\delta_e)$ in an AB pair. This renders AB interactions negative-sum: the total payoff in an AB encounter becomes $-(1-\alpha)\delta_e < 0$ for $0 \le \alpha < 1$, similar to AA (total $-2\delta_s$) and BB pairs (total $-2\delta_n$).

Using routine calculations as in the Materials & Methods section in the main text, we obtain the possible individual optimum x^* and social optimum x^s for the equilibrium gun ownership rate as follows (see Fig. S10):

$$\begin{cases} x^* = \dfrac{b_g - c_g + p_a(\delta_n + \alpha\delta_e)}{p_a\big(\delta_n + \delta_s - (1-\alpha)\delta_e\big)}, \\ x^s = \dfrac{b_g - c_g + p_a\big(2\delta_n - (1-\alpha)\delta_e\big)}{2p_a\big(\delta_n + \delta_s - (1-\alpha)\delta_e\big)}. \end{cases} \tag{S27}$$

Since both x^* and x^s must lie between zero and one to be meaningful, this leads to the following cases depending on the sign of $b_g - c_g$.

For costly individual gun ownership ($b_g - c_g < 0$), we have

$$x^* = \begin{cases} 0 & 0 \le p_a \le \dfrac{c_g - b_g}{\delta_n + \alpha\delta_e} \\ \dfrac{b_g - c_g + p_a(\delta_n + \alpha\delta_e)}{p_a\big(\delta_n + \delta_s - (1-\alpha)\delta_e\big)} & \dfrac{c_g - b_g}{\delta_n + \alpha\delta_e} < p_a \le 1 \end{cases} \tag{S28}$$

and the social optimum

$$x^s = \begin{cases} 0 & 0 \le p_a \le \dfrac{c_g - b_g}{2\delta_n - (1-\alpha)\delta_e} \\ \dfrac{b_g - c_g + p_a\big(2\delta_n - (1-\alpha)\delta_e\big)}{2p_a\big(\delta_n + \delta_s - (1-\alpha)\delta_e\big)} & \dfrac{c_g - b_g}{2\delta_n - (1-\alpha)\delta_e} < p_a \le 1 \end{cases} \tag{S29}$$

For beneficial individual gun ownership ($b_g > c_g$, we have

$$x^* = \begin{cases} 1 & 0 \le p_a \le \dfrac{b_g - c_g}{\delta_s - \delta_e} \\ \dfrac{b_g - c_g + p_a(\delta_n + \alpha\delta_e)}{p_a\big(\delta_n + \delta_s - (1-\alpha)\delta_e\big)} & \dfrac{b_g - c_g}{\delta_s - \delta_e} < p_a \le 1 \end{cases}, \tag{S30}$$

and the social optimum

$$x^s = \begin{cases} 1 & 0 \le p_a \le \dfrac{b_g - c_g}{2\delta_s - (1-\alpha)\delta_e} \\ \dfrac{b_g - c_g + p_a\left(2\delta_n - (1-\alpha)\delta_e\right)}{2p_a\left(\delta_n + \delta_s - (1-\alpha)\delta_e\right)} & \dfrac{b_g - c_g}{2\delta_s - (1-\alpha)\delta_e} < p_a \le 1 \end{cases} \tag{S31}$$

The scenario with neutral gun ownership cost, $b_g - c_g = 0$, resembles the case $b_g - c_g < 0$, except that when $p_a = 0$, any value of $x \in [0, 1]$, constitutes a neutral equilibrium for both the individual and social optima.

With these analytical expressions, it is straightforward to demonstrate how the discounting parameter α impacts the social dilemma of overarming. Simple algebraic calculations show that both the individual optimum x^* and the social optimum x^s are monotonically increasing functions of the discounting parameter α for both $b_g - c_g < 0$ (cf. Figs. S10A-C) and $b_g - c_g > 0$ (cf. Figs. S10D-F). In contrast, the misalignment between individual and social interests, measured by their difference $x^* - x^s$, is monotonically decreasing in α, indicating that increasingly negative-sum AB interactions can exacerbate the social dilemma of overarming up to the threshold

$$\alpha_c = \frac{c_g - b_g + \delta_e - 2\delta_n}{\delta_e}. \tag{S32}$$

For α below this critical value of α_c (i.e., $\alpha < \alpha_c$), the social optimum x^s drops to zero gun ownership when $p_a = 1$ regardless of the sign of $b_g - c_g$ (see Figs. S10A and S10D).

That said, for $\alpha < \alpha_c$, the social optimum can become zero for sufficiently large values of p_a, whereas the individual optimum of gun ownership rate remains positive. This means that the misalignment between individual and social interests – and thus overarming – can persist even when the advantage of having a gun against non-owner, δ_e, is strongly discounted.

Taken together, our main conclusion regarding overarming remains qualitatively unchanged under payoff asymmetry that leads to negative-sum AB interactions: the individually optimal equilibrium still exceeds the socially optimal level (even when the latter becomes zero), thereby preserving the misalignment between individual and social interests for sufficiently large values of p_a.

Accounting for behavioral heterogeneity among gun owners: fair-use vs. always-use in confrontations Our base model only considers one type of gun owner, who always resorts to using a

gun in any confrontation (always-use gun owners, denoted by A_1), and therefore does not account for heterogeneity in gun owners' propensity to escalate conflicts to gun use. However, some gun owners may choose to use guns at their discretion based on subjective motivations, and may not necessarily escalate to gun use, especially when the other party is unarmed non-owner (denoted by B). This consideration gives rise to a third behavioral type of fair-use gun owners (denoted by A_0): they neither seek an advantage over non-owners nor wish to be disadvantaged against other gun owners. The social payoff matrix $\mathbf{M}_s$ for confrontations involving guns is

$$\begin{array}{c} \\ A_0 \\ A_1 \\ B \end{array} \begin{array}{c} \begin{array}{ccc} A_0 & A_1 & B \end{array} \\ \begin{pmatrix} -\delta_n & -\delta_s & -\delta_n \\ -\delta_s & -\delta_s & \delta_e \\ -\delta_n & -\delta_e & -\delta_n \end{pmatrix} \end{array}. \tag{S33}$$

At first glance, interactions with fair-use gun owners (A_0) yield equal payoffs for both parties, analogous to the fair-minded Tit-for-Tat strategy in classic social dilemmas of cooperation. In this spirit, the presence of A_0 can mitigate the fear of being disadvantaged among non-owners (B, analogous to AllC) and neutralize the potential advantage δ_e of gun owners who always use guns in any conflict, even against an unarmed opponent (A_1, analogous to AllD).

Weighting $\mathbf{M}_s$ by the provocation rate p_a and combining it with the individual payoff matrix $\mathbf{M}_o$ for gun ownership,

$$\begin{array}{c} \\ A_0 \\ A_1 \\ B \end{array} \begin{array}{c} \begin{array}{ccc} A_0 & A_1 & B \end{array} \\ \begin{pmatrix} b_g - c_g & b_g - c_g & b_g - c_g \\ b_g - c_g & b_g - c_g & b_g - c_g \\ 0 & 0 & 0 \end{pmatrix} \end{array}, \tag{S34}$$

we obtain the overall payoff matrix $\mathbf{M} = \mathbf{M}_o + p_a\mathbf{M}_s$:

$$\mathbf{M} = \begin{array}{c} \\ A_0 \\ A_1 \\ B \end{array} \begin{array}{c} \begin{array}{ccc} A_0 & A_1 & B \end{array} \\ \begin{pmatrix} b_g - c_g - p_a d_n & b_g - c_g - p_a d_s & b_g - c_g - p_a d_n \\ b_g - c_g - p_a d_s & b_g - c_g - p_a d_s & b_g - c_g + p_a d_e \\ -p_a d_n & -p_a d_e & -p_a d_n \end{pmatrix} \end{array}. \tag{S35}$$

Let x_i represent the fraction of each type, for $i \in \{1, 2, 3\}$ corresponding to A_0, A_1, and B respectively. The population composition lies on the simplex defined by the vector $\mathbf{x} = [x_1, x_2, x_3]^\top$

with $\sum_{i=1}^{3} x_i = 1$ and $0 \leq x_i \leq 1$ for $i = 1, 2, 3$. The three-strategy replicator dynamics are given given by:

$$\dot{x}_i = x_i\big((\mathbf{Mx})_i - \mathbf{x} \cdot \mathbf{Mx}\big), \tag{S36}$$

leading to nontrivial dynamics within the simplex (see Fig. S11).

Of interest is to analyze the existence of all possible equilibria (including corner, edge, and interior equilibria) and their stability. Intuitive approaches such as invasion analysis (comparing the average payoff of a rare mutant against a resident population) and formal Jacobian analysis can inform the local stability of an equilibrium. For brevity, we state the results without going into the details.

All pure strategies (the corner equilibria A_0, A_1, and B) are unstable for most scenarios, with a few exceptions. The corner B is stable when gun ownership is individually costly, namely when

$$b_g - c_g < -p_a(\delta_e + \delta_n). \tag{S37}$$

And fair-use gun owner A_0 is stable only if individual gun ownership is perceived as beneficial, i.e., when $b_g - c_g > 0$.

The A_1–B edge equilibrium, a coexistence state between A_1 and B, exists and is stable when

$$-p_a(\delta_e + \delta_n) < b_g - c_g < p_a(\delta_s - \delta_e), \tag{S38}$$

and its coordinates are formally the same as in the original base model consisting of strategies A_1 and B:

$$x_1^* = 0, \quad x_2^* = \frac{b_g - c_g + p_a(\delta_n + \delta_e)}{p_a(\delta_n + \delta_s)}, \quad x_3^* = 1 - x_2^*.$$

Such a coexistence equilibrium can exist across all scenarios, regardless of whether $b_g - c_g < 0$ or not, as long as the condition (S38) is satisfied (cf. Fig. S11A-C).

Most interestingly, every point on the A_0–B edge is an edge equilibrium when $b_g - c_g = 0$ (neutral gun ownership cost), since A_0 and B are neutral. Moreover, as shown in Fig. S11b, the segment of this A_0–B edge is stable when the fraction of A_0 exceeds the threshold

$$x_1 > \frac{\delta_e + \delta_n}{\delta_e + \delta_s} \tag{S39}$$

The presence of fair-use gun owners A_0 can act like Tit-for-Tat players that shepherd non-owners (B, analogous to AllC), and together this polymorphism resists the invasion by always-use gun

owners(A_1, analogous to AllD). This is the intuition behind the Rock-Paper-Scissors-like cycling yet with damping as shown in Fig. S11.

An interior equilibrium exists when

$$0 < b_g - c_g < p_a(\delta_s - \delta_e), \tag{S40}$$

and is given by

$$x_2^* = \frac{b_g - c_g}{p_a(\delta_s - \delta_e)}, \quad x_1^* = \frac{\delta_e + \delta_n}{\delta_e + \delta_s}(1 - x_2^*), \qquad x_3^* = \frac{\delta_s - \delta_n}{\delta_e + \delta_s}(1 - x_2^*).$$

In this case, the interior equilibrium is an unstable saddle, separating trajectories that are attracted either to A_0 or to the coexistence edge equilibrium on the A_1–B edge (see Fig. S11).

Fig. S11 shows representative evolutionary dynamics of the three types within the simplex.

As shown in Fig. S11A, for $b_g - c_g < 0$, the dynamics resembles a Rock-Paper-Scissors-like cycle, but with damping due to costly gun ownership: the population moves away from states with abundant A_1 toward higher prevalence of A_0, which in turn facilitates B; however, B remains vulnerable to invasion by A_1, and the system ultimately converges to a mixed equilibrium on the A_1–B edge.

As shown in Fig. S11B, for $b_g - c_g = 0$, the polymorphism consisting of A_0 and B is stable against invasion by A_1 provided that the fraction of A_0 exceeds $(\delta_e + \delta_n)/(\delta_e + \delta_s)$. Otherwise, the population converges to the same edge equilibrium as in Fig. S11A.

As shown in Fig. S11C, for $b_g - c_g > 0$, A_0 can be a dominant strategy in addition to the mixed equilibrium on the A_1–B edge, leading to bistability, with the two basins of attraction separated by an unstable interior equilibrium.

In conclusion, this extended analysis shows that introducing a third type of fair-use gun owners can facilitate de-escalation under certain parameter regimes. Together, these extensions under alternative assumptions enrich and strengthen the robustness of our modeling analysis presented in the main text.

Table S3: Empirical justification for model parameters related to gun ownership and social aggression dynamics.

Model parameter	Qualitative and quantitative support from prior studies or survey reports
p_a: **Provocation rate/confrontation probability** *Likelihood of gun-related interpersonal confrontations or perceived threat.*	• **Prevalence of threat**: A 2017 Pew survey found 23% of U.S. adults say they or a family member have been threatened or intimidated with a gun (75). This indicates a significant incidence of interpersonal gun threats.<br>• **Perceived risk/fear**: Nearly 48% of Americans reported in 2019 that they worry at least somewhat about being the victim of a mass shooting (76), reflecting a high perceived threat of violent confrontation. Similarly, fear of crime has driven many to arm themselves for protection (77).<br>• **Reported gun violence (confrontation) rates**: Justice Department statistics show the rate of nonfatal firearm violence is around 2 per 1,000 persons annually in recent years (78). In other words, each year about 0.2% of Americans reported a robbery or assault where the offender has a gun. While not every citizen will face a gun confrontation, the number of gun-involved altercations is substantial; for example, over 160,000 injuries or deaths from firearm assaults occurred annually in the late 1980s (79). These data underscore that confrontations involving guns, or the fear of them, are a real concern of the population.

b_g: **Individual benefit of gun ownership** *Personal gains (safety, utility, peace of mind, recreation) from owning a gun.*	• **Protection and safety**: Protection is the dominant motive for U.S. gun owners. In 2023, 72% of gun owners told Pew researchers that personal protection is a major reason they own a firearm (*80*). Correspondingly, 81% of owners say they feel safer having a gun in the home (*80*). This highlights the psychological benefit of security; owning a gun provides peace of mind and a sense of safety against crime.<br>• **Deterrence and empowerment**: Surveys indicate many owners believe a gun is a useful defensive tool. By 2021, 88% of gun owners (up from 67% in 2000) cited "protection against crime" as a reason for ownership (*77*). The perceived benefit is that being armed equalizes power in threatening situations, deterring criminals and reducing fear of being victimized.<br>• **Recreation and other benefits**: Beyond self-defense, gun ownership confers recreational and intangible benefits. A majority of owners enjoy shooting as a hobby (71% say they enjoy owning a gun) (*80*). National surveys show about 70% of owners use firearms for target shooting and $\tilde{5}$6% for hunting (*77*). These survey results indicate guns provide sport and cultural value. These personal and social benefits (e.g., sport, collecting, tradition) contribute to the overall utility b_g for individuals who choose to own guns.

**c_g: Individual cost of gun ownership** *Personal risks and downsides (physical, financial, psychological) of owning a gun.*	• **Increased injury and death risk**: Extensive public-health research shows that keeping a gun is associated with higher risk of harm. A 2014 meta-analysis found individuals with firearm access have two times higher odds of being a homicide victim and three times higher odds of suicide than those without guns ([20]). In short, a gun in the home substantially raises the likelihood of deadly outcomes, whether from violence, accidents, or self-harm ([81]).<br>• **Accidents and unintentional harm**: Gun ownership also carries risk of unintentional shootings (especially with improper storage). In 2022, there were 463 unintentional firearm deaths in the U.S. ([81]), and many more nonfatal accidental shootings (often involving children). An estimated 4.6 million children live in homes with a loaded, unlocked gun ([81]), creating potential for tragic mishaps. These numbers illustrate the ever-present safety costs (injury, death, trauma) that come with owning firearms.<br>• **Mental and financial costs**: Owning a gun can impose psychological stress or financial burdens. Some gun owners acknowledge worrying about gun-related accidents or theft (though only 12% report worrying about having a gun at home ([80]). Additionally, the monetary cost of purchasing firearms, ammunition, training, and secure storage can be significant. While harder to quantify, these factors contribute to c_g. Notably, for every case of defensive gun use, far more people report being threatened or harmed with guns ([82]), implying that the net personal benefit of gun ownership is not without trade-offs in risk.

**δ_s: Social cost of shootout (armed confrontation)** *Interpersonal and societal cost when both parties in a confrontation are armed (e.g., potential for shootouts and escalation).*	• **Escalation to lethal violence**: When two individuals are armed during a confrontation, the encounter is far more likely to escalate into a deadly exchange of gunfire. Research suggests that an armed person is actually at risk in such scenarios: In a case-control study in Philadelphia, those possessing a gun were 4.5 times more likely to be shot in an assault compared to those not carrying a gun (*56*). In other words, a tense altercation can quickly turn into a bilateral shootout, greatly increasing the probability that someone (or both parties) gets seriously injured or killed.<br>• **Mutual risk and bystander harm**: Armed confrontations (two-gun scenarios) create crossfire that endangers not only the participants but also bystanders. For example, road rage incidents illustrate this social cost: by 2023, U.S. road rage shootings (often involving armed drivers on both sides) doubled from a few years prior, with someone shot or wounded in a road-rage encounter every 18 hours in 2023 (*83*). These incidents sometimes result in both shooter and victim being shot, or innocent third parties (passengers, nearby drivers) being hurt. Thus, δ_s – the societal cost of two armed people clashing – includes the heightened chance of a firefight, potential injury to both combatants, and collateral damage to the public (*82*). In essence, an armed society faces greater risks that routine disputes turn into lethal events.

**δ_n: Social cost of non-gun confrontation** *Harm resulting from confrontations when no guns are involved (e.g., fistfights).*	• **Lower lethality of unarmed fights**: When conflicts occur without firearms, they are far less likely to be deadly. Disputes are often settled with verbal aggression or at worst fists/knives, leading to injury but seldom death. For instance, an analysis of Chicago assault data found that knife attacks outnumbered gun attacks 2.3 to 1, yet the fatality rate per 100 knife attacks was only about one-fifth that of gun attacks (*84*). Even when focusing only on serious woundings, gun assaults were 2.5 times more likely to be fatal than knife assaults (*84*). This illustrates that without guns, conflicts tend to cause less permanent harm – an attacker's intent to kill often cannot be realized as easily with other weapons or physical force.<br><br>• **Typical outcomes of unarmed altercations**: Socially, δ_n can include injuries from brawls or psychological trauma from threats, but the scope of harm is usually limited. A bar fight or a domestic dispute with no gun present might result in bruises, perhaps stabbings in extreme cases, but rarely the mass casualties or quick deaths that guns can inflict. Statistics show most aggravated assaults in the U.S. do not involve firearms, and those altercations largely end with the parties surviving (albeit sometimes with injuries) (*79*). In sum, the cost of a non-gun confrontation, while not trivial (medical care, emotional distress, etc.), is considerably lower than when firearms are in play. Society bears fewer lasting consequences because unarmed or knife fights are less likely to produce fatalities or irreversible damage.

**δ_e: Concession cost of being unarmed vs. an armed opponent** *Disadvantage or harm to a person who is unarmed when confronting someone with a gun (power asymmetry).*	• **Fear and power asymmetry**: The psychological toll of being unarmed against an armed threat is significant. The unarmed individual often has no choice but compliance or escape; any resistance could be met with lethal force. This fear of being helpless in the face of an armed attacker is a documented driver of gun ownership (e.g., 81% of U.S. gun owners say being armed makes them feel safer (*80*). Qualitatively, victims describe the experience of facing a gun while unarmed as terrifying and disempowering. Even if no shots are fired, the armed person holds all the power in the situation. The cost δ_e is thus measured in both tangible harm and intimidation: an unarmed person can be robbed, assaulted or controlled with near-impunity by an armed aggressor. As Pew research notes, about 1 in 4 Americans have felt this vulnerability – 23% have either personally or via family been at the receiving end of gun threats (*75*).<br><br>• **Victimization rates**: Empirical evidence shows that unarmed individuals frequently find themselves on the losing side of confrontations with armed aggressors. In national surveys, people report being threatened with a gun far more often than they report defending themselves with one. One study found Americans were three times more likely to have been threatened or harmed by a gun than to have used a gun in self-defense (*82*). Likewise, analysis of crime surveys indicates nine times as many people are criminally victimized with a gun than are protected by a gun in any given year (*82*). These figures highlight the severe power imbalance and high personal cost of being unarmed in a confrontation where the other party has a gun.

Sources: References 75-84 as listed in the main text.

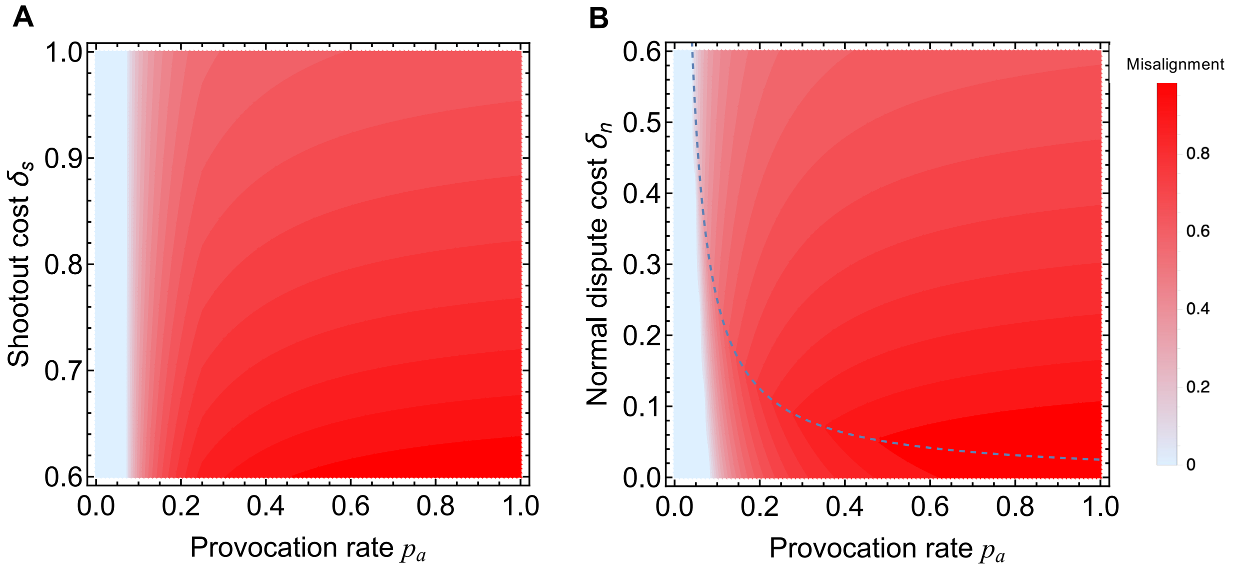

Figure S1: **Social dilemma of overarming impacted by the perceived cost of shootout, δ_s and by the perceived cost of normal dispute, δ_n.** (**A**) Misalignment level is depicted as a heatmap over the parameter space (p_a, δ_s). (**B**) Similar heatmap as in (**A**) but over the parameter space (p_a, δ_n). The blue regions in (**A**) and (**B**) highlight the combinations of model parameters yielding zero gun acquisitions under individual self-interest, which also exactly aligns with social optimum. The dashed line in (**B**) marks the boundary below which the social optimum for gun acquisitions remains zero. In general, the levels of misalignment decrease as δ_s and δ_n increase, particularly when both optima are nonzero. Parameters: $b_g = 0.1$, $c_g = 0.15$; (**A**): $\delta_e = 0.6$, $\delta_n = 0.1$; (**B**): $\delta_s = 1$, $\delta_e = 0.6$.

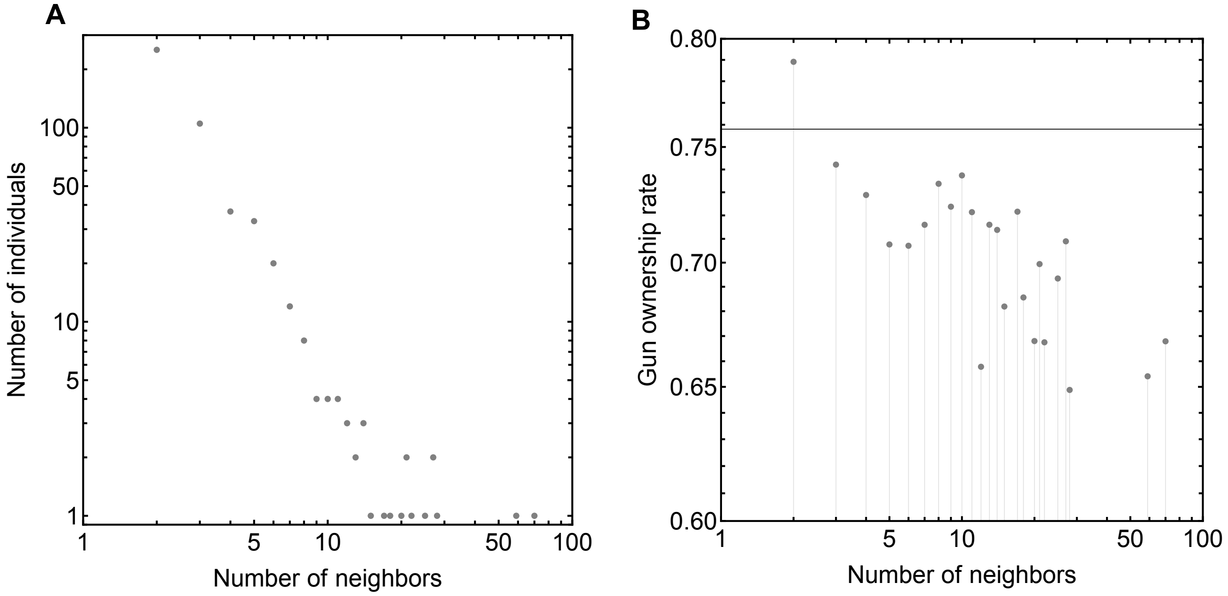

Figure S2: **The impact of degree on gun acquisitions in heterogeneous social networks.** Individuals at the central nodes are less inclined to own guns compared to those at the periphery nodes who have fewer neighbors. (**A**) Degree distribution of a degree-heterogenous model network. (**B**) A scatter plot illustrating the relationship between gun acquisitions and the number of an individual's neighbors. The horizontal line marks the network's average gun ownership rate at 75.8%. Parameters: network size 500, average degree 4, $K = 0.1$, $b_g = 0.1$, $c_g = 0.15$, $p_a = 0.5$, $\delta_s = 1$, $\delta_e = 0.6$, $\delta_n = 0.1$.

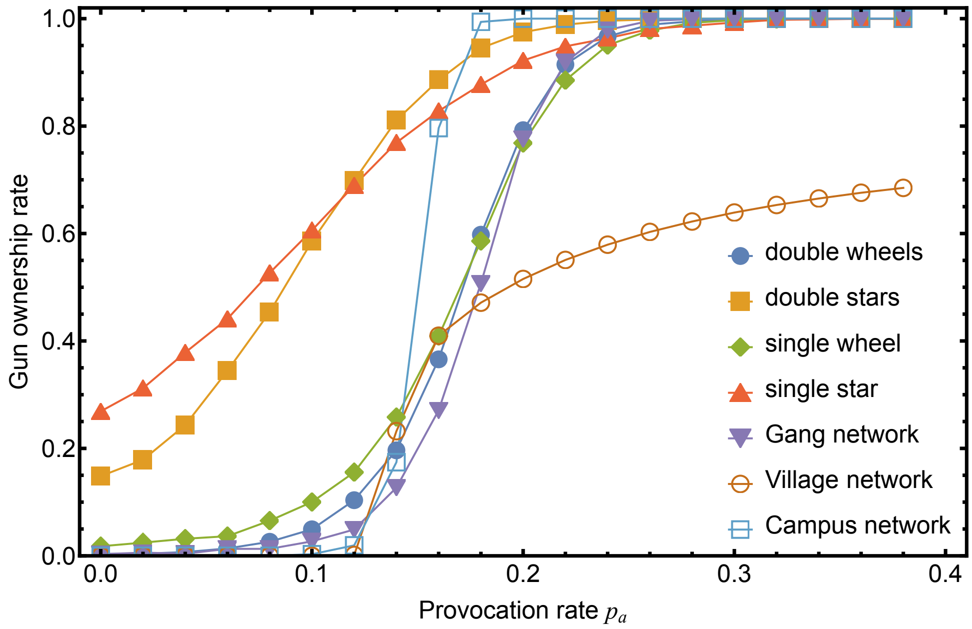

Figure S3: **The impact of network heterogeneity on gun acquisitions.** The presence of star-like structure, together with clustering, can facilitate sharp transitions from zero to universal gun ownership. Comparative network snapshots reveal that the real gang and campus networks exhibit topological characteristics that bear resemblance to stylized structures like the single wheel and double wheels. In comparison, the star and double stars are even stronger amplifiers of gun acquisitions. Parameters: $K = 0.1$, $b_g = 0.1$, $c_g = 0.15$, $\delta_s = 1$, $\delta_e = 0.6$, $\delta_n = 0.1$. The real networks are detailed as in the main text. For comparison, we examine stylized structures including the single star and wheel, double stars, and double wheels, all of which consist of 30 nodes each. Initial conditions are 50% A's and 50% B's, and results are averaged over 5000 independent runs using synchronous updating.

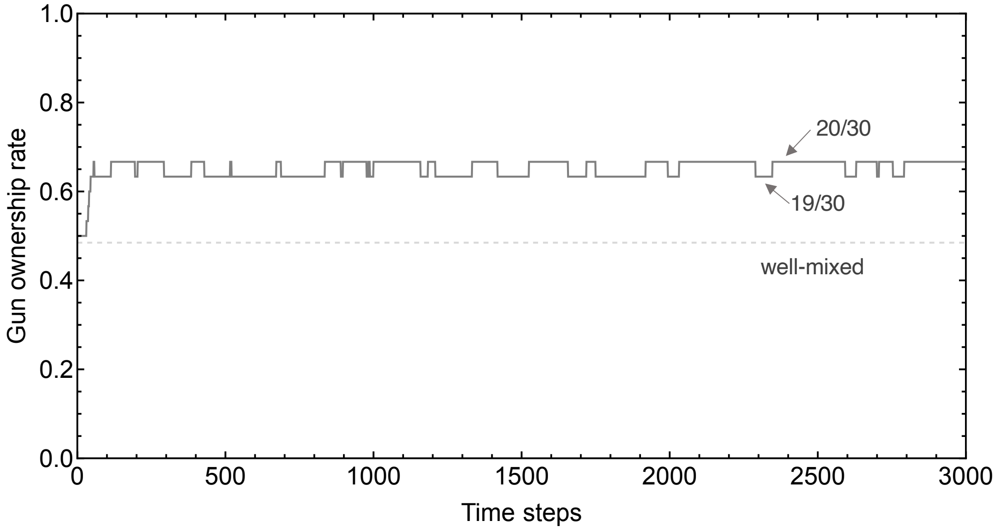

Figure S4: **Time series of gun acquisitions on a star graph.** Starting from an equal mix of A and B individuals, the system enters into oscillatory dynamics (i.e., oscillating between $19/30$ and $20/30$) in which the leaf nodes are frozen in their strategic choices while the center alternates probabilistically between A (owning a gun) and B (not owning a gun). The dashed line is the individual self-interest equilibrium in well-mixed populations. Parameters: $K = 1 \times 10^{-6}$, $p_a = 0.3$, $b_g = 0.1$, $c_g = 0.15$, $\delta_s = 1$, $\delta_e = 0.6$, $\delta_n = 0.1$, star network size 30, initial conditions: 50% A's and 50% B's, and asynchronous updating.

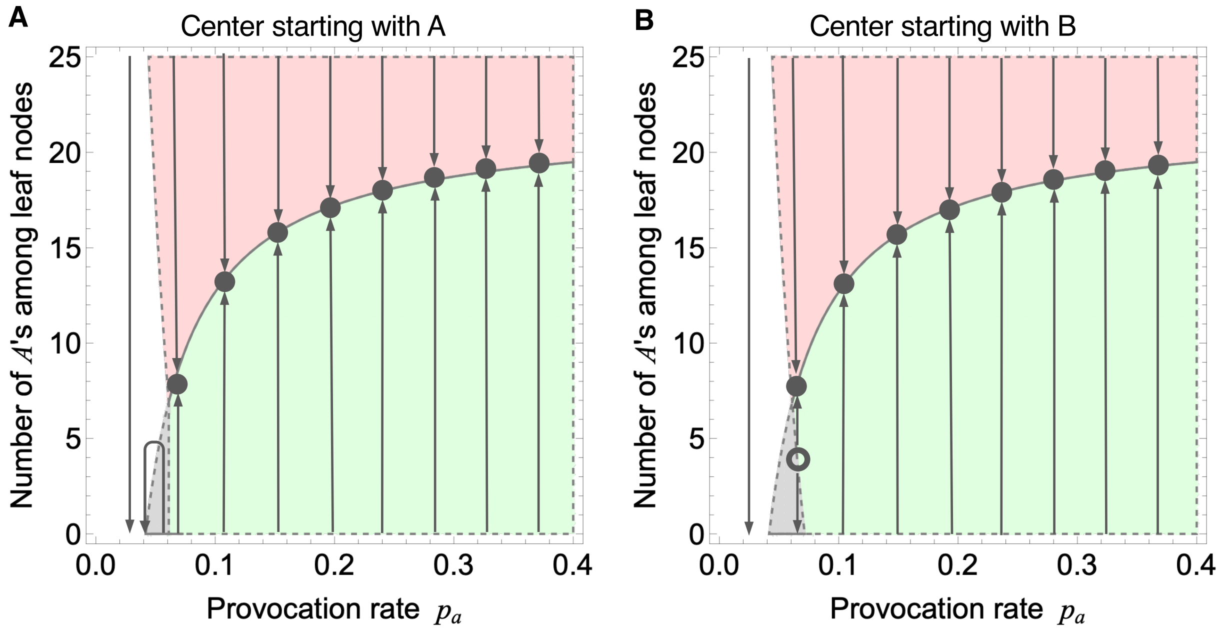

Figure S5: **Spread of gun acquisitions (*A* individuals) in star graphs.** The spread dynamics depends on whether the center (hub) starts with A. (**A**) Shown is the attraction of basin, when the center starts with A, as a function of the relative provocation rate, p_a, and the number of A's in leaf nodes.The colors of the parameter regions suggest whether the number of A's among leaf nodes can increase or not, indicated by the arrows. The green region indicates that a center A has a higher payoff than periphery B's and that a center B has a lower payoff than periphery A's. The red region indicates that a center A has a lower payoff than periphery B's and that a center B has a lower payoff than periphery A's. The most interesting region is the grey region in which the single center A first spreads to periphery nodes but once reaching a critical number of A's, all A's go extinct. Unless the critical number is an integer, the system will end up oscillatory dynamics (around the floor value of the filled circles) where the center alternates between A and B while the leaf nodes are frozen with their states. (**B**) The phase diagram depicts similar dynamics as in (**A**), except that the center starts with B. Moreover, there exists an interesting bistability behavior, as indicated by the empty circle in (**B**). Parameters: we consider perfect rationality, $K \to 0$. $b_g = 0.1$, $c_g = 0.15$, $\delta_s = 1$, $\delta_e = 0.6$, $\delta_n = 0.1$, star network size 30.

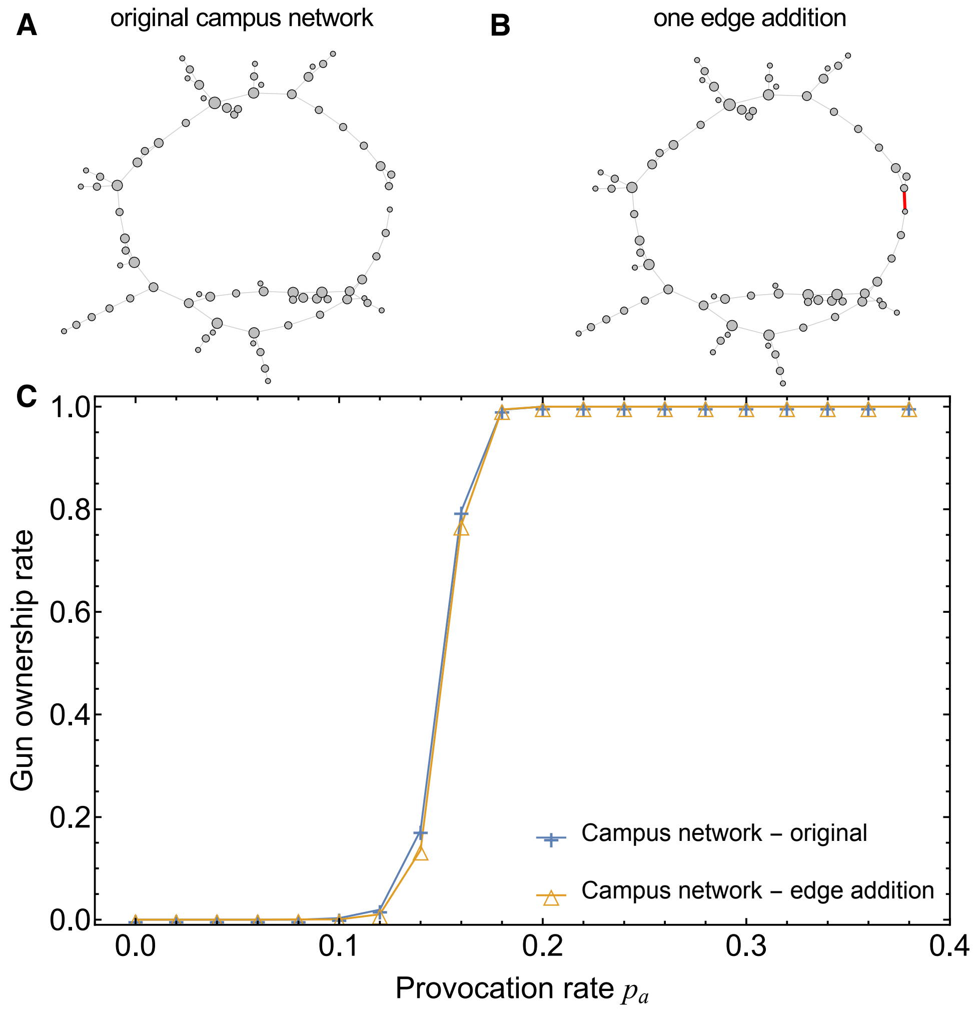

Figure S6: **Even small changes in social network structure can have an impact on gun acquisitions.** (**A** and **B**) show the original campus network in comparison to the where one edge is added between a pair of nodes having the largest path length. The added one edge, highlighted in red in (**B**), connects two leaf nodes situated in two local star motifs. (**C**) depicts the equilibrium gun acquisition rate as function of the provocation rate p_a, suggesting that the network with one edge added has higher critical p_a transitioning from zero to full acquisition rate with moderately lower gun acquisition within the transition. This result indicates that small changes in the social network structure can have an impact on lowering gun acquisition for intermediate ranges of p_a. The parameters used are the same as in the main Fig. 3.

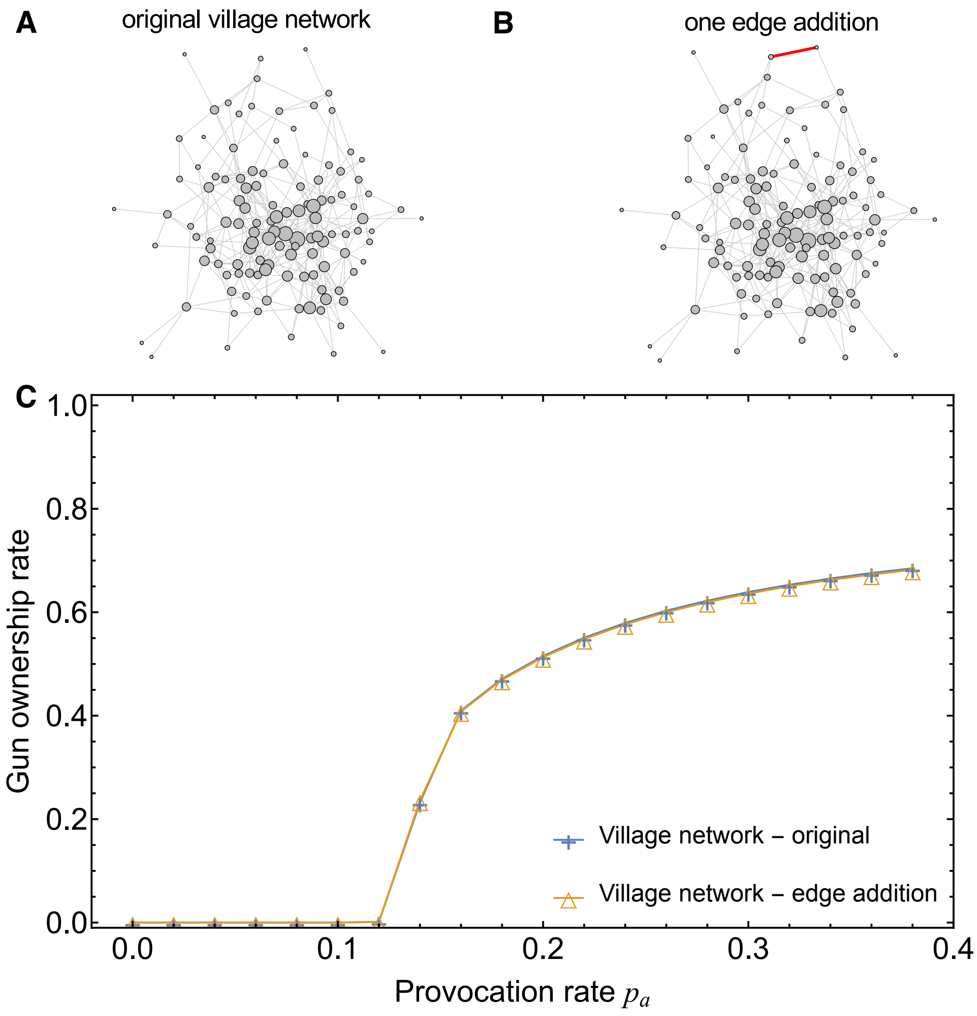

Figure S7: **Even small changes in social network structure can have an impact on gun acquisitions.** (**A** and **B**) show the original village network in comparison to the where one edge is added between a pair of nodes having the largest path length. The added one edge, highlighted in red in (**B**), connects two periphery nodes situated in two local star-like motifs. (**C**) depicts the equilibrium gun acquisition rate as function of the provocation rate p_a, suggesting that the network with one edge added has a noticeably lower gun acquisition for large values of p_a. This result indicates that small, targeted changes in the social network structure can have an impact on lowering gun acquisition. The parameters used are the same as in the main Fig. 3.

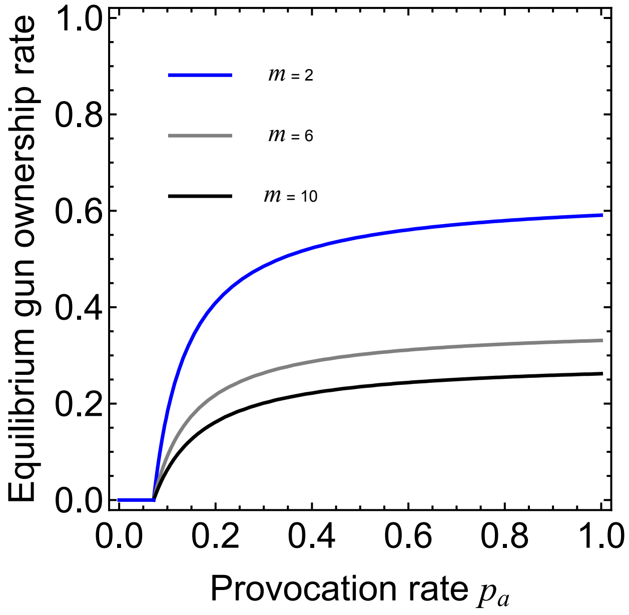

Figure S8: **Impact of group interactions on gun acquisition.** Shown is the equilibrium gun ownership rate as a function of the provocation rate p_a for different group sizes $m = 2, 6, 10$. Increases in group size beyond two result in the presence of simultaneous multiple guns in the group and thus neutralize their advantage in group settings. Consequently, this leads to lower gun acquisition. Parameters are the same as in the main Fig. 1A, except for the group size m as indicated in the plot legend.

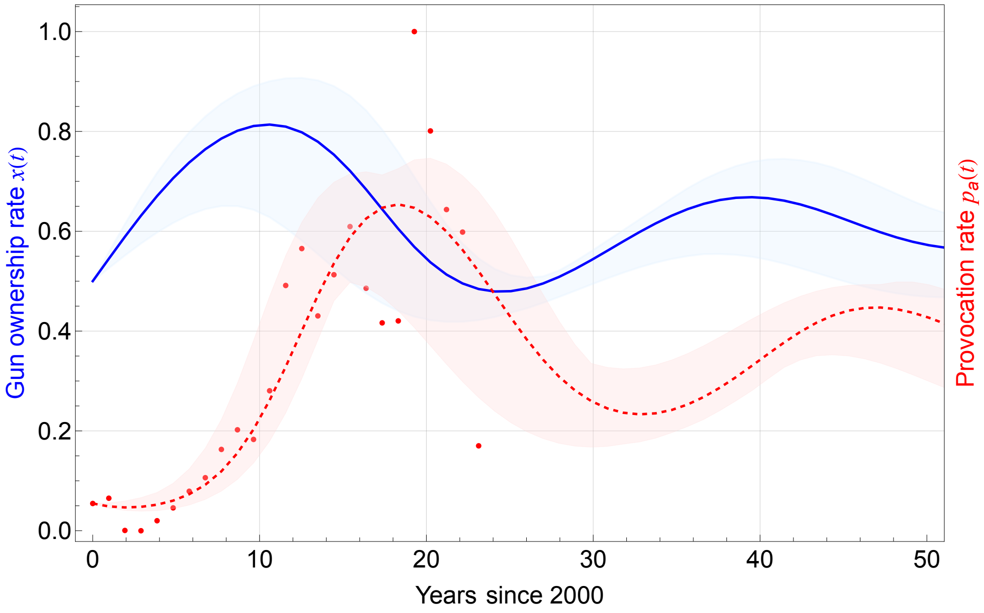

Figure S9: **Model-based insights into the feedback dynamics between perceived risk and gun ownership in the United States.** We use gun sales as a proxy for the extent of perceived threat in our coevolutionary model of gun acquisition, $x(t)$, and threat perception, $p_a(t)$. The dots represent normalized annual gun sales, and the solid lines are best-fitted curves obtained from our model, with the corresponding shaded areas representing uncertainty quantified at the 95% confidence levels. Our model provides insights into the interplay between perceived threat and gun acquisition. See Table S2 for model parameter estimations.

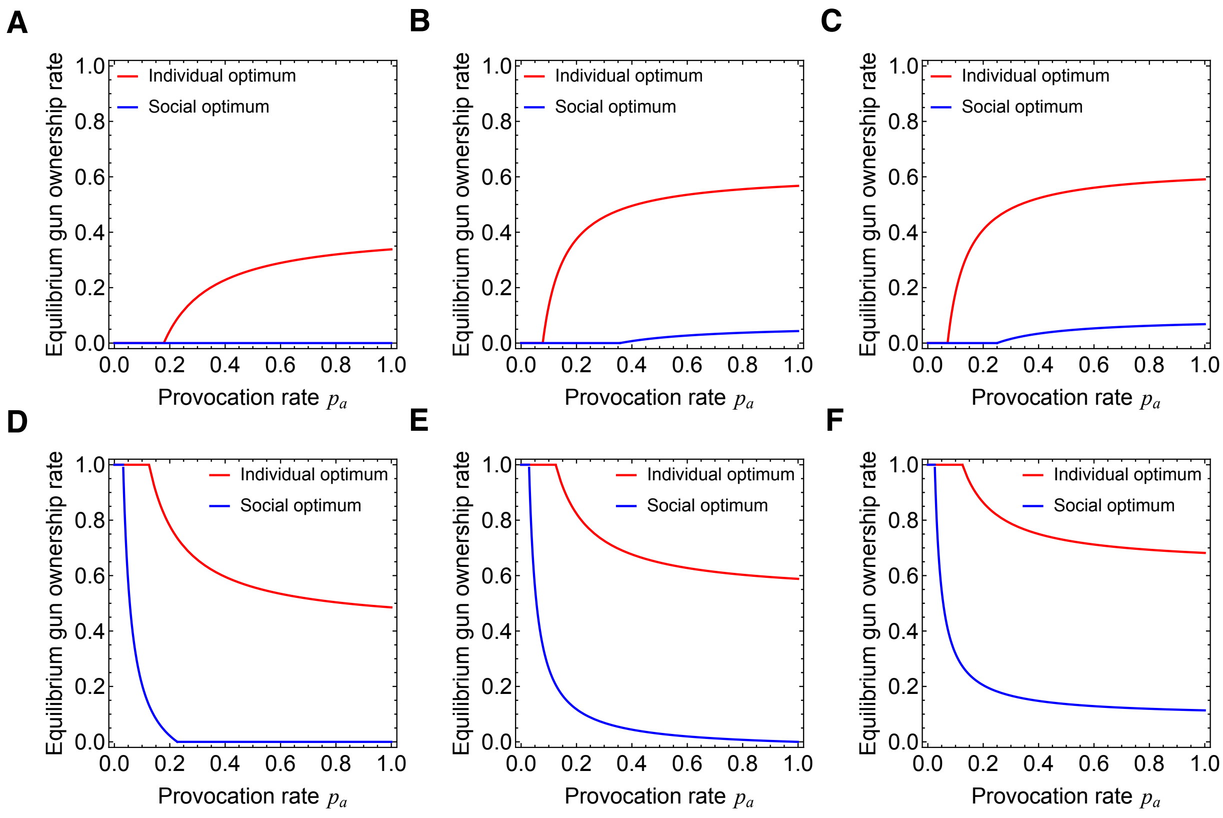

Figure S10: **Impact of negative-sum interactions in AB encounters.** Shown are the individual optimum (ESS) versus the social optimum of the equilibrium gun ownership rate as a function of the provocation rate p_a in well-mixed populations for both $b_g < c_g$ (**A** - **C**) and $b_g > c_g$ (**D** - **F**). These plots how these optima are affected when A receives a discounted payoff $\alpha\delta_e$ $(0 < \alpha < 1)$ when against B (whose concession cost remains $-\delta_e$), while all other payoffs remain the same as in the base model (i.e., $\alpha = 1$). For both $b_g < c_g$ and $b_g > c_g$, introducing the discounting parameter α reduces both the socially optimal gun ownership rate and the individual optimum rate. Moreover, the social optimum can become zero at $p_a = 1$ when $\alpha < \alpha_c = (c_g - b_g + \delta_e - 2\delta_n)/\delta_e$. The misalignment between individual and social interests, and thus overarming, still persists when discounting the advantage of a gun owner against a non-owner. Parameters: $\delta_s = 1$, $\delta_e = 0.6$, $\delta_n = 0.1$; (**A** - **C**): $b_g = 0.1$, $c_g = 0.15$; (**D** - **F**): $b_g = 0.15$, $c_g = 0.1$; (**A** and **D**): $\alpha = 0.3$; (**B**): $\alpha = 0.9$; (**E**): $\alpha = 7/12$; (**C** and **F**): $\alpha = 1$.

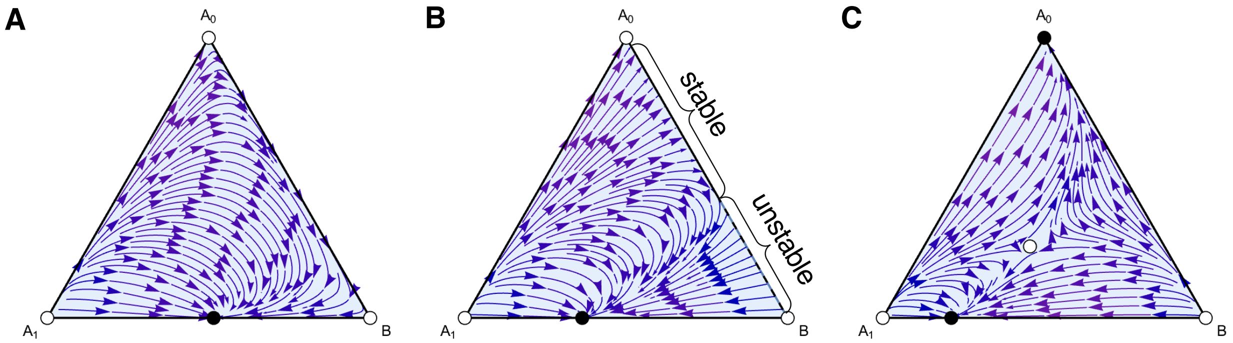

Figure S11: **Evolutionary dynamics of gun ownership in the simplex with third type of fair-use gun owner, alongside the original two types.** We denote by A_0 fair-use gun owners who do not wish to use guns to gain an advantage but who resort to using a gun only if the other party uses a gun in conflict. The original two types are denoted by A_1 (gun owners who always use their gun in a confrontation, i.e., "always escalate" to a gunfight) and B (non-owners), respectively. We plot the vector fields of the replicator dynamics for the three strategies in the simplex and use circles to represent equilibria, including corner, edge, and interior equilibria. Filled circles indicate stable equilibria, whereas open circles indicate unstable equilibria. (**A**): for $b_g < c_g$, the resulting dynamics resembles a Rock-Paper-Scissors cycle, albeit with damping due to costly gun ownership. The population moves away from states with abundant A_1 toward a higher prevalence of A_0, which in turn facilitates the takeover by B; however, B remains vulnerable to invasion by A_1, and the system ultimately converges to a mixed equilibrium on the A_1–B edge. (**B**): for $b_g = c_g$, the polymorphism consisting of A_0 and B is stable against invasion by A_1 provided that the fraction of A_0 exceeds $(\delta_e + \delta_n)/(\delta_e + \delta_s)$. Otherwise, the population converges to the same equilibrium on the A_1–B edge as in panel **a**. (**C**): for $b_g > c_g$, there can exist an interior equilibrium that is an unstable saddle, separating trajectories that are attracted either to the corner A_0 or to the same mixed equilibrium on the A_1–B edge as in (**A**) and (**B**). parameters: $\delta_s = 1$, $\delta_e = 0.6$, $\delta_n = 0.1$, $p_a = 0.3$; (**A**): $b_g = 0.1$, $c_g = 0.15$; (**B**): $b_g = 0.1$, $c_g = 0.1$; (**C**): $b_g = 0.15$, $c_g = 0.1$.

REFERENCES

1. Pew Research Center, "For Most U.S. Gun Owners, Protection Is the Main Reason They Own a Gun," Technical Report by Pew Research Center (2023); www.pewresearch.org/wp-content/uploads/sites/20/2023/08/PP_2023.08.16_gun-owners_REPORT.pdf.

2. "Gun Ownership by State," *wisevoter* (2026); https://wisevoter.com/state-rankings/gun-ownership-by-state.

3. "Gun Ownership by Country 2026," *World Population Review* (2026); https://worldpopulationreview.com/country-rankings/gun-ownership-by-country.

4. N. Buttrick, Protective gun ownership as a coping mechanism. *Perspect. Psychol. Sci.* **15**, 835–855 (2020).

5. W. Stroebe, N. P. Leander, A. W. Kruglanski, Is it a dangerous world out there? The motivational bases of American gun ownership. *Pers. Soc. Psychol. Bull.* **43**, 1071–1085 (2017).

6. J. J. Bélanger, N. P. Leander, M. Agostini, J. Kreienkamp, W. Stroebe, Passion for guns and beliefs in a dangerous world: An examination of defensive gun ownership. *Aggress. Behav.* **50**, e22146 (2024).

7. J. Carlson, *Citizen-Protectors: The Everyday Politics of Guns in an Age of Decline* (Oxford Univ. Press, 2015).

8. P. J. Cook, J. J. Donohue, Saving lives by regulating guns: Evidence for policy. *Science* **358**, 1259–1261 (2017).

9. A. Winkler, *Gunfight: The Battle Over the Right to Bear Arms in America* (WW Norton and Company, 2011).

10. P. J. Cook, J. Ludwig, *Guns in America: National Survey on Private Ownership and Use of Firearms* (US Department of Justice, Office of Justice Programs, National Institute of Justice, 1997).

11. N. Buttrick, J. Mazen, Historical prevalence of slavery predicts contemporary American gun ownership. *PNAS Nexus* **1**, pgac117 (2022).

12. A. Filindra, "Race, rights, and rifles: The origins of the NRA and contemporary gun culture" in *Race, Rights, and Rifles* (University of Chicago Press, 2023).

13. L. Berkowitz, A. LePage, Weapons as aggression-eliciting stimuli. *J. Pers. Soc. Psychol.* **7**, 202–207 (1967).

14. B. J. Bushman, Guns automatically prime aggressive thoughts, regardless of whether a 'good guy' or 'bad guy' holds the gun. *Soc. Psychol. Pers. Sci.* **9**, 727–733 (2018).

15. B. J. Bushman, C. A. Anderson, J. Allen, "General aggression model" in *The International Encyclopedia of Media Psychology* (John Wiley & Sons, 2020), pp. 1–9.

16. S. Shepherd, A. C. Kay, Guns as a source of order and chaos: Compensatory control and the psychological (dis) utility of guns for liberals and conservatives. *J. Assoc. Consum. Res.* **3**, 16–26 (2018).

17. D. Yamane, The sociology of US gun culture. *Sociol. Compass* **11**, e12497 (2017).

18. D. Yamane, P. Yamane, S. L. Ivory, Targeted advertising: Documenting the emergence of gun culture 2.0 in guns magazine, 1955–2019. *Palgrave Commun.* **6**, 61 (2020).

19. D. M. Studdert, Y. Zhang, E. E. Holsinger, L. Prince, A. F. Holsinger, J. A. Rodden, G. J. Wintemute, M. Miller, Homicide deaths among adult cohabitants of handgun owners in California, 2004 to 2016: A cohort study. *Ann. Intern. Med.* **175**, 804–811 (2022).

20. A. Anglemyer, T. Horvath, G. Rutherford, The accessibility of firearms and risk for suicide and homicide victimization among household members: A systematic review and meta-analysis. *Ann. Intern. Med.* **160**, 101–110 (2014).

21. D. Hemenway, S. J. Solnick, Children and unintentional firearm death. *Inj. Epidemiol.* **2**, 1–6 (2015).

22. M. Siegel, C. S. Ross, C. King, A new proxy measure for state-level gun ownership in studies of firearm injury prevention. *Inj. Prev.* **20**, 204–207 (2014).

23. A. R. Andrés, K. Hempstead, Gun control and suicide: The impact of state firearm regulations in the United States, 1995–2004. *Health Policy* **101**, 95–103 (2011).

24. G. Kleck, *Targeting Guns: Firearms and their Control* (Routledge, 2017).

25. C. C. Lanfear, R. Bucci, D. S. Kirk, R. J. Sampson, Inequalities in exposure to firearm violence by race, sex, and birth cohort from childhood to age 40 years, 1995-2021. *JAMA Netw. Open* **6**, e2312465 (2023).

26. J. J. Donohue, A. Aneja, K. D. Weber, Right-to-carry laws and violent crime: A comprehensive assessment using panel data and a state-level synthetic control analysis. *J. Empir. Leg. Stud.* **16**, 198–247 (2019).

27. L. H. Monteiro, More guns, less crime? A dynamical systems approach. *Appl. Math Comput.* **369**, 124804 (2020).

28. G. Kleck, M. Gertz, Armed resistance to crime: The prevalence and nature of self-defense with a gun. *J. Crim. Law Criminol.* **86**, 1995 (1973).

29. R. Taylor, A game theoretic model of gun control. *Int. Rev. Law Econ.* **15**, 269–288 (1995).

30. M. Siegel, C. S. Ross, C. King III, The relationship between gun ownership and firearm homicide rates in the United States, 1981–2010. *Am. J. Public Health* **103**, 2098–2105 (2013).

31. D. Wodarz, N. L. Komarova, Dependence of the firearm-related homicide rate on gun availability: A mathematical analysis. *PLOS ONE* **8**, e71606 (2013).

32. “General Methodology,” *Gun Violence Archive* (2023); www.gunviolencearchive.org/methodology.

33. “Past Summary Ledgers” *Gun Violence Archive* (2026); www.gunviolencearchive.org/past-tolls.

34. “Mass Shootings by Country 2026,” *World Population Review* (2026); https://worldpopulationreview.com/country-rankings/mass-shootings-by-country.

35. J. R. Lott, *More Guns, Less Crime: Understanding Crime and Gun Control Laws* (University of Chicago Press, 2013).

36. C. Sathya, F. L. Dreier, M. L. Ranney, To prevent gun injury, build better research. *Nature* **610**, 30–33 (2022).

37. H. M. Mialon, T. Wiseman, The impact of gun laws: A model of crime and self-defense. *Econ. Lett.* **88**, 170–175 (2005).

38. A. V. Papachristos, A. A. Braga, D. M. Hureau, Social networks and the risk of gunshot injury. *J. Urban Health* **89**, 992–1003 (2012).

39. A. V. Papachristos, C. Wildeman, E. Roberto, Tragic, but not random: The social contagion of nonfatal gunshot injuries. *Soc. Sci. Med.* **125**, 139–150 (2015).

40. B. Green, T. Horel, A. V. Papachristos, Modeling contagion through social networks to explain and predict gunshot violence in Chicago, 2006 to 2014. *JAMA Intern. Med.* **177**, 326–333 (2017).

41. D. M. Hureau, T. Wilson, H. M. Jackl, J. Arthur, C. Patterson, A. V. Papachristos, Exposure to gun violence among the population of Chicago community violence interventionists. *Sci. Adv.* **8**, eabq7027 (2022).

42. F. Fu, D. N. Rockmore, Too little, too late—A dynamical systems model for gun-related violence and intervention. *Appl. Math Comput.* **467**, 128495 (2024).

43. D. G. Rand, M. A. Nowak, Human cooperation. *Trends Cogn. Sci.* **17**, 413–425 (2013).

44. M. Jusup, P. Holme, K. Kanazawa, M. Takayasu, I. Romić, Z. Wang, S. Geček, T. Lipić, B. Podobnik, L. Wang, W. Luo, T. Klanjšček, J. Fan, S. Boccaletti, M. Perc, Social physics. *Phys. Rep.* **948**, 1–148 (2022).

45. Y. Zhang, F. Fu, T. Wu, G. Xie, L. Wang, A tale of two contribution mechanisms for nonlinear public goods. *Sci. Rep.* **3**, 2021 (2013).

46. A. Glaubitz, F. Fu, Evolutionary branching and consistency in human cooperation: The interplay of incentives and volunteerism in addressing collective action dilemmas. arXiv:2303.01622 [physics.soc-ph] (2023).

47. M. D. Anestis, C. J. Bryan, Threat perceptions and the intention to acquire firearms. *J. Psychiatr. Res.* **133**, 113–118 (2021).

48. H. Kahn, *On Thermonuclear War* (Princeton Univ. Press, 1960).

49. C. Cohen, L. Rinot Levavi, A game-theory-based approach to promoting health policy among minorities. *Int. J. Environ. Res. Public Health* **20**, 4335 (2023).

50. H. Ohtsuki, C. Hauert, E. Lieberman, M. A. Nowak, A simple rule for the evolution of cooperation on graphs and social networks. *Nature* **441**, 502–505 (2006).

51. M. O. Jackson, L. Yariv, Diffusion of behavior and equilibrium properties in network games. *Am. Econ. Rev.* **97**, 92–98 (2007).

52. D. Centola, The spread of behavior in an online social network experiment. *Science* **329**, 1194–1197 (2010).

53. U. Alvarez-Rodriguez, F. Battiston, G. F. de Arruda, Y. Moreno, M. Perc, V. Latora, Evolutionary dynamics of higher-order interactions in social networks. *Nat. Hum. Behav.* **5**, 586–595 (2021).

54. K. Parker, J. M. Horowitz, R. Igielnik, J. B. Oliphant, A. Brown, “America’s Complex Relationship with Guns,” Technical Report by Pew Research Center (2017); www.pewresearch.org/social-trends/2017/06/22/the-demographics-of-gun-ownership.

55. “Gun Ownership by State,” *wisevoter* (2023); https://wisevoter.com/state-rankings/gun-ownership-by-state.

56. C. C. Branas, T. S. Richmond, D. P. Culhane, T. R. Ten Have, D. J. Wiebe, Investigating the link between gun possession and gun assault. *Am. J. Public Health* **99**, 2034–2040 (2009).

57. D. Hemenway, D. Azrael, M. Miller, Gun use in the United States: Results from two national surveys. *Inj. Prev.* **6**, 263–267 (2000).

58. D. Hemenway, Survey research and self-defense gun use: An explanation of extreme overestimates. *J. Crim. Law Criminol.* **87**, 1430 (1997).

59. D. G. Watts, S. H. Strogatz, Collective dynamics of 'small-world' networks. *Nature* **393**, 440–442 (1998).

60. N. A. Christakis, J. H. Fowler, Social contagion theory: Examining dynamic social networks and human behavior. *Stat. Med.* **32**, 556–577 (2013).

61. G. Szabó, C. Töke, Evolutionary prisoner's dilemma game on a square lattice. *Phys. Rev. E* **58**, 69–73 (1998).

62. K. Descormiers, C. Morselli, Alliances, conflicts, and contradictions in Montreal's street gang landscape. *Int. Crim. Justice Rev.* **21**, 297–314 (2011).

63. N. A. Christakis, J. H. Fowler, Social network sensors for early detection of contagious outbreaks. *PLOS ONE* **5**, e12948 (2010).

64. F. Fu, N. A. Christakis, J. H. Fowler, Dueling biological and social contagions. *Sci. Rep.* **7**, 43634 (2017).

65. D. A. Kim, A. R. Hwong, D. Stafford, D. A. Hughes, A. J. O'Malley, J. H. Fowler, N. A. Christakis, Social network targeting to maximise population behaviour change: A cluster randomised controlled trial. *Lancet* **386**, 145–153 (2015).

66. L. J. Kolbe, School gun violence in the United States. *J. Sch. Health* **90**, 245–253 (2020).

67. M. Landa-Blanco, H. Cheon, L. G. R. Flores, C. Spohn, C. M. Katz, Violence in Honduras from 2008 to 2018. *Inj. Prev.* **26**, 191–193 (2020).

68. F. C. Santos, J. M. Pacheco, Scale-free networks provide a unifying framework for the emergence of cooperation. *Phys. Rev. Lett.* **95**, 098104 (2005).

69. S. W. Webster, E. C. Connors, B. Sinclair, The social consequences of political anger. *J. Polit.* **84**, 1292–1305 (2022).

70. M. Fügenschuh, F. Fu, Overcoming vaccine hesitancy by multiplex social network targeting: An analysis of targeting algorithms and implications. *Appl. Netw. Sci.* **8**, 67 (2023).

71. X. Wang, F. Fu, Eco-evolutionary dynamics with environmental feedback: Cooperation in a changing world. *Europhys. Lett.* **132**, 10001 (2020).

72. A. R. Morral, R. Smart, Better data, less gun violence. *Science* **377**, 1471 (2022).

73. J. M. Smith, G. R. Price, The logic of animal conflict. *Nature* **246**, 15–18 (1973).

74. A. Traulsen, J. M. Pacheco, M. A. Nowak, Pairwise comparison and selection temperature in evolutionary game dynamics. *J. Theor. Biol.* **246**, 522–529 (2007).

75. "Guns in America: Attitudes and Experiences of Americans," *Pew Research Center* (2017); www.pewresearch.org/social-trends/2017/06/22/americas-complex-relationship-with-guns/.

76. "Nearly Half in U.S. Fear Being the Victim of a Mass Shooting," *Gallup* (2019); https://news.gallup.com/poll/266681/nearly-half-fear-victim-mass-shooting.aspx.

77. "Gun Owners Increasingly Cite Crime as Reason for Ownership," *Gallup* (2021); https://news.gallup.com/poll/357329/gun-owners-increasingly-cite-crime-reason-ownership.aspx.

78. Bureau of Justice Statistics, "Trends and Patterns in Firearm Violence, 1993–2023" (2024); https://bjs.ojp.gov/library/publications/trends-and-patterns-firearm-violence-1993-2023.

79. Bureau of Justice Statistics, "Firearms and Crimes of Violence: Selected Findings From National Statistical Series" (1994); https://bjs.ojp.gov/content/pub/pdf/fcvsfnss.pdf.

80. "For Most U.S. Gun Owners, Protection Is the Main Reason They Own a Gun," *Pew Research Center* (2023); www.pewresearch.org/politics/2023/08/16/for-most-u-s-gun-owners-protection-is-the-main-reason-they-own-a-gun/.

81. Center for Gun Violence Solutions, Johns Hopkins Bloomberg School of Public Health, *Firearm Violence in the United States* (2023); https://publichealth.jhu.edu/center-for-gun-violence-solutions/research-reports/gun-violence-in-the-united-states.

82. Center for American Progress, *Debunking the "Guns Make Us Safer" Myth* (2015); www.americanprogress.org/article/debunking-the-guns-make-us-safer-myth/.

83. Everytown Research and Policy, *Road Rage Shootings Remain Alarmingly High* (2024); https://everytownresearch.org/road-rage-shootings-remain-alarmingly-high/.

84. Office of Justice Programs, *Is Gun Control Likely to Reduce Violent Killings?* National Criminal Justice Reference Service; www.ojp.gov/ncjrs/virtual-library/abstracts/gun-control-likely-reduce-violent-killings.
